# No detectable remission-specific remodeling of intestinal monocyte and macrophage metabolic transcriptional programs after adalimumab in Crohn’s disease: A patient-level reanalysis of a longitudinal single-cell atlas

**DOI:** 10.1371/journal.pone.0356953

**Published:** 2026-11-11

**Authors:** Ayma Waqar

**Affiliations:** 1 University of Pennsylvania School of Engineering and Applied Science, Philadelphia, Pennsylvania, United States of America; 2 The Children’s Hospital of Philadelphia, Philadelphia, Pennsylvania, United States of America; University of Colombo Faculty of Medicine, SRI LANKA

## Abstract

We examined whether remission after adalimumab in Crohn’s disease (CD) was associated with differential change in intestinal monocyte and macrophage metabolic transcription. We reanalyzed the corrected public TAURUS atlas using 19 site-matched biopsy pairs from 15 patients (10 remission, 5 nonremission). Remission required at least two of Harvey–Bradshaw Index <5, Nancy index ≤1 and no ulceration; post-treatment sampling occurred at least eight weeks after treatment began. Seven programs were evaluated using an outcome-blind continuum, patient-level paired changes and exact permutation tests. Twelve eligible pairs, including five of six nonremission pairs, came from terminal ileum. No primary contrast survived correction (14 tests; minimum P = 0.397; all adjusted P = 0.987). The glycolysis-level difference in change was −0.005 score units (Welch 95% CI −0.052 to 0.042). Alternative scoring, trajectories and composition analyses also yielded no corrected program contrast. Published TAURUS benchmarks were reproduced within the discovery data. Batch explained 41.5% of patient outcome variation; within-batch inference relied on nine patients and 40 label allocations (minimum adjusted P = 0.350). Donor pseudobulk analysis tested 7,092 expressed genes with paired edgeR models allowing batch-specific time effects; none survived correction (minimum adjusted P = 0.894). None of 17 metabolic transcript modules survived correction of 34 directional and mixed tests (minimum adjusted P = 0.134). Including all 26 CD pairs and varying cell thresholds yielded no corrected program contrast. Empirical 80%-power detection thresholds were approximately 1.4–1.6 SD per primary endpoint, describing detection sensitivity without establishing equivalence or excluding effects of that size. Sparse colonic remission biopsies, batch imbalance and missing individual treatment metadata constrain interpretation. These findings concern RNA programs; they do not establish absence of metabolic remodeling, treatment causality or metabolic-flux effects.

## Introduction

Anti-tumor necrosis factor (anti-TNF) antibodies remain a mainstay of treatment for Crohn’s disease (CD), but many patients do not reach remission, and the tissue features that distinguish treatment outcomes are incompletely understood [1,2]. Single-cell studies have associated anti-TNF resistance in CD with an inflammatory module of IgG plasma cells, inflammatory mononuclear phagocytes, activated T cells and stromal cells [1], and inflammatory bowel disease (IBD) more broadly with inflammatory monocytes and an IL-1-driven stromal-neutrophil axis [3,4].

Intestinal macrophages are continuously replenished from circulating monocytes, which mature through intermediate states into resident macrophages, a sequence often called the monocyte waterfall [5,6]. Human small-intestinal macrophages show a comparable spectrum from recently recruited monocyte-like cells to long-lived resident macrophages [7]. In inflamed CD and ulcerative colitis (UC), inflammatory monocytes and IL1B-expressing macrophages accumulate alongside resident-like macrophages [1,8,9]. An expression continuum is therefore a reasonable descriptive model for monocyte and macrophage states in biopsies, but inflammatory and resident-like macrophages may be alternative branches rather than consecutive steps of one path.

Metabolic programs are linked to macrophage function. Inflammatory activation is commonly accompanied by increased glycolysis and pentose phosphate pathway activity, whereas resident and regulatory macrophages rely more on oxidative metabolism and lipid handling [10,11]. In CD, glycolytic activation of CD14-positive intestinal macrophages supports inflammatory output through membrane TNF [12], and in experimental models the efficacy of anti-TNF antibodies requires macrophage IL-10 signaling [13]. Recent reviews propose macrophage immunometabolism as a therapeutic axis in IBD [14,15]. Whether these programs change differently in patients who do and do not reach remission on anti-TNF therapy has not been tested in human tissue with patient-level inference. Because transcript abundance does not measure substrate flux, such an analysis can address only transcriptional organization.

The TAURUS study profiled 216 intestinal biopsies from patients with CD or UC before and after adalimumab [2]. In CD it reported that a higher pretreatment proportion of C1Q-high IL1B-low macrophages was associated with later remission, that the myeloid compartment contracted after treatment in remission, and that S100A8/A9-high monocytes in nonremission increased *TNFRSF1B*, *TREM1* and cathepsin expression after treatment [2]. A pediatric study likewise described broad cellular changes after anti-TNF therapy [16]. Neither study examined longitudinal change in metabolic transcriptional programs along the monocyte-to-macrophage continuum.

We asked whether the post-minus-pre change in seven prespecified metabolic programs differs between patients with CD who are and are not in remission after adalimumab, with the patient as the inferential unit. This is a treatment-by-outcome contrast rather than a baseline prediction analysis. We do not claim novelty for the TAURUS cell-state annotations or abundance findings; instead, we reproduce several of them as positive controls to calibrate the sensitivity of the patient-level pipeline. We also evaluate robustness to the trajectory method, the scoring method, cellular composition, tissue site and statistical assumptions, and we quantify the effects this cohort could detect.

## Materials and methods

### Study design and data sources

This is a secondary analysis of de-identified public data from the TAURUS atlas (GEO GSE282122) [2]. We used the corrected processed release (Zenodo record 14007626; concept DOI 10.5281/zenodo.13768607), which supersedes an earlier release with incorrect metadata: the myeloid object myeloid_final.h5ad, the paired-sample list paired_sample_list.csv and the source S1, S5, S7 and S8 Tables. The source study enrolled 38 biologic-naive adults with CD or UC who were starting adalimumab and 3 healthy controls, and sampled up to five intestinal sites before and after treatment. Biopsies were cryopreserved, predigested with EDTA, dissociated with Liberase TM and DNase, and profiled with 10x Genomics Chromium 3-prime v3 or v3.1 chemistry; reads were processed with Cell Ranger 3.1.0, and cells with fewer than 500 genes, more than 60% mitochondrial reads or predicted doublets were removed [2]. The released myeloid object (30,858 cells and 33,075 genes, raw counts) was generated by the source investigators by subclustering with Harmony integration [17]; we used its cell-state labels and Harmony coordinates without re-annotation.

### Data integrity and reproduction of the original analysis

File checksums matched the upstream MD5 values and the archived SHA-256 manifest. We ran 47 programmatic integrity checks (S1 Table), all of which passed: all 45,481,461 stored count values were nonnegative integers; per-cell total counts equalled matrix row sums; cell, barcode, sample and gene identifiers were unique and mutually consistent; Harmony coordinates were finite and row-aligned; and sample-level fields were constant within each biopsy and agreed with source S1 Table for all 216 biopsies (patient, site, treatment, sequencing batch, chemistry, sex, inflammation score and disease duration in months). Recorded age differed between the two sources only for the 3 healthy controls, who were not used for inference. Remission labels were re-derived from the published clinical criteria for all 16 patients with CD and agreed in every case. The archived analysis scripts (Zenodo 10.5281/zenodo.21829569, version 0.1.0) were rerun in their pinned Python environment; all 27 archived result tables, including the diffusion embedding and the 14 primary tests, were regenerated with a maximum absolute numeric difference of 0. An independent reimplementation of the primary tests agreed to within 1.2e-16.

### Analysis population, outcomes and clinical context

The corrected paired list defines 110 biopsies forming 55 exact patient-by-site pre/post pairs, of which 26 pairs from 15 patients had CD. Remission was defined by the source investigators per patient at follow-up as at least two of Harvey-Bradshaw Index (HBI) <5, Nancy histological index ≤1 and absence of macroscopic ulceration; escalation to another advanced therapy for uncontrolled disease was classed as nonremission [2]. Post-treatment biopsies were taken at least 8 weeks after starting adalimumab and up to about 1.5 years later, with all patients on therapy at resampling; the source cohort summary reports a mean interval of 11 (SD 4.2) months in CD, but patient-level intervals, dosing and concomitant medications were not released. As prespecified, a site pair was eligible when both biopsies contained at least 30 monocytes or macrophages, with 50 cells as a sensitivity threshold. Patient characteristics were taken from source S1 Table, (Table 1). Every eligible pair shared the same sequencing batch label and 10x chemistry at both time points.

**Table 1:** Characteristics of the 15 patients with Crohn’s disease in the primary analysis.

Characteristic	Remission (n = 10)	Nonremission (n = 5)
Patients, n	10	5
Age at baseline, years, median (IQR)	33 (28-42)	38 (37-41)
Female, n	3	3
Disease duration, months, median (IQR)	50 (15-120)	124 (98-228)
Montreal location L1/L2/L3, n	0/3/7	3/0/2
Montreal behaviour B1(incl. B1p)/B2/B3, n	7/2/1	3/2/0
Baseline HBI, median (IQR)	9 (6-12)	10 (9-13)
Follow-up HBI, median (IQR)	0 (0-2)	10 (10-11)
Baseline macroscopic ulceration, n	8	5
Follow-up macroscopic ulceration, n	2	5
Baseline Nancy index, median (IQR)	1 (0-2)	2 (2-4)
Follow-up Nancy index, median (IQR) [missing]	0 (0-1) [0]	1 (0-2) [1]
Treatment failure (escalation), n	0	5
Eligible site pairs, n (TI/AC/DC/rectum)	13 (7/3/2/1)	6 (5/1/0/0)
Excluded site pairs (<30 cells), n	6	1
Sequencing batch of eligible pairs (1/2/3/4), n	1/5/5/2	4/0/2/0
Baseline inflammation score, median (IQR)	7.82 (6.99-8.08)	8.89 (7.51-9.06)
Monocyte/macrophage cells, pre / post	2,056 / 814	1,037 / 2,194
All post-QC cells, pre / post	57,746 / 62,911	24,706 / 28,140

Clinical variables are from source S1 Table (Thomas et al.); remission was defined at follow-up by at least two of Harvey-Bradshaw Index (HBI) <5, Nancy index ≤1 and no macroscopic ulceration, with escalation classed as nonremission. Disease duration is in months. Montreal location L1 ileal, L2 colonic, L3 ileocolonic; behaviour B1 nonstricturing nonpenetrating (B1p with perianal disease), B2 stricturing, B3 penetrating. Site abbreviations: TI terminal ileum, AC ascending colon, DC descending colon. Inflammation score is the source study’s transcriptomic score. Cell counts refer to eligible pairs. IQR, interquartile range.

### Outcome-blind continuum and trajectory analyses

The original continuum was built from cells labelled Mono or Macro (15,707 cells; four states: S100A8/A9-high monocytes, S100A8/A9-high TNF-high IL6-positive monocytes, C1Q-high IL1B-high macrophages and C1Q-high IL1B-low macrophages). A 30-nearest-neighbor graph on the released Harmony coordinates was used to compute diffusion pseudotime (DPT) with Scanpy 1.12.3 (seed 1729) [18,19], rooted in the S100A8/A9-high monocyte with the largest FCN1, S100A8, S100A9, VCAN and CCR2 minus C1QA, C1QB, C1QC, APOE and MERTK expression contrast. Treatment and outcome labels were not used. Because the root markers are themselves monocyte and macrophage markers, we also defined an independent maturation score from markers not used for rooting (CD163, MRC1, MAF, SELENOP, CD209 and FOLR2 minus S100A12, LYZ, SELL, CD300E and EREG).

Four further orderings were evaluated, chosen to address branching, embedding dependence and method dependence [20]. First, official Slingshot 2.20.0 (Bioconductor 3.23, R 4.6.1) [21] was run on a SingleCellExperiment built directly from the h5ad counts with the released 50-dimensional Harmony coordinates, released state labels as clusters and S100A8/A9-high monocytes as the start cluster. Sensitivity runs used 10 or 20 Harmony dimensions, the omega option, and annotation-free Louvain (k = 30) and k-means (k = 10) clusters with the start cluster containing the DPT root cell. Second, Palantir 1.4.1 [22] was run on the same Harmony coordinates from the same root cell. Third, to test whether metabolic genes shaped the embedding, we rebuilt the continuum after excluding all 707 mapped metabolic-program genes and all mitochondrial and ribosomal protein genes from highly variable gene selection, followed by principal component analysis, Harmony integration by biopsy (harmonypy 2.1.0), a 30-nearest-neighbor graph and DPT from the original root. Fourth, a rooted geodesic ordering from the previous revision was regenerated for comparison. Pseudotime is interpreted as an expression ordering, not as lineage tracing or elapsed time.

### Metabolic transcriptional programs and scoring

Seven programs were fixed before outcome modeling: MSigDB Hallmark 2020 glycolysis, oxidative phosphorylation (OXPHOS), fatty acid metabolism and cholesterol homeostasis [23]; Reactome 2022 citric acid (TCA) cycle (R-HSA-71403) and pentose phosphate pathway (R-HSA-71336) [24]; and KEGG 2021 Human lysosome [25]. The archived manifest contains 795 gene-by-program entries, 710 distinct source genes, 707 distinct genes present in the TAURUS object and 694 distinct genes with nonzero variance among monocytes and macrophages (S2 Table). Thus the figure of 707 genes in the original submission referred to mapped genes, and 710 to source genes. 67 genes belonged to more than one program, and 14 of 21 program pairs overlapped (for example, OXPHOS and the TCA cycle), so the seven scores are not independent. The OXPHOS set contains no MT-prefixed genes.

Original scores were the mean, across program genes, of per-gene standardized log-normalized expression (counts per 10,000, log1p, standardized across all 15,707 cells). As a second, rank-based method we ran the official UCell 2.16.0 package [26] on raw counts of the full transcriptome (33,075 genes), so that genes were ranked within each cell against all expressed genes; the default rank cutoff of 1,500 was primary and cutoffs of 800 and 3,000 were sensitivity analyses. Thirty size-matched random gene sets per program, drawn from genes detected in at least 1% of cells, were scored with UCell as a competitive reference [27]. For sub-pathway resolution we prespecified a panel of 31 rate-limiting or representative enzymes and regulators (for example *HK2*, *PFKFB3*, *LDHA*, *G6PD*, *CPT1A*, *HMGCR* and *ACOD1*) and per-cell mitochondrial and ribosomal read percentages as dissociation-stress indicators.

### Patient-level outcomes and statistical analysis

For each biopsy and program we computed the level (mean cell score) and the slope (ordinary least-squares coefficient of score on pseudotime). Pre-treatment values were subtracted from post-treatment values within each site pair, and pairs were averaged within patient so that each patient contributed one value per endpoint. The primary estimand was the mean change in the 10 remission patients minus the mean change in the 5 nonremission patients. Two-sided exact P values enumerated all C(15,10) = 3,003 allocations of outcome labels. Benjamini-Hochberg correction [28] was applied across the 14 primary tests (7 levels and 7 slopes) and within each sensitivity family; an exact single-step maximum-statistic family-wise adjustment was also computed [29], and the effective number of independent endpoints was 6 by the eigenvalue method [30].

The statistical methods were audited before reporting (S1 Appendix). Simulations that resampled the observed patient residuals showed that the original stratified percentile bootstrap intervals undercovered (mean coverage 0.89, minimum 0.83, nominal 0.95), whereas Welch t intervals achieved a mean coverage of 0.94. We therefore report Welch 95% confidence intervals and retain the original bootstrap intervals for comparison. Because outcome groups are observational, exchangeability of labels cannot be assumed. When the smaller nonremission group had the larger variance, the unstudentized permutation test had a simulated type I error of up to 0.14 at a nominal 0.05; a studentized (Welch statistic) exact permutation test [31,32] reduced the maximum to 0.095, and both P values are reported (S7 Fig; S7 Table). Standardized differences are Hedges g with the pooled patient-level standard deviation.

Within-group post-minus-pre changes were tested with exact sign-flip tests over all sign assignments. These describe change during treatment and are not causal estimates, because there is no untreated comparison group. No equivalence test was performed, because no margin could be justified independently of the observed data [33]; we report estimation intervals and empirical detection limits instead.

### Sensitivity analyses added after peer review

All analyses in this section were specified after peer review and are exploratory. Branch-specific inference used cells with Slingshot curve weight or Palantir fate probability of at least 0.5 and required at least 20 such cells in both biopsies of a pair; families with fewer than 3 patients per group were not tested. Composition was addressed by within-state analyses (at least 10 cells of the state in both biopsies) and by a Kitagawa decomposition [34] of each pair’s change into a within-state component (state means changing at the average composition) and a composition component (state proportions changing at the average state means). Common pseudotime support was the interval covered by the 5th to 95th percentiles of every eligible biopsy. Nonlinear profiles were summarized by Theil-Sen slopes, Spearman correlations and a binned functional contrast (sum of squared Welch statistics across pseudotime quintile bins, assessed by exact permutation). Alternative patient-level models comprised heteroskedasticity-consistent (HC3) regression, baseline-adjusted analysis of covariance (post on remission and baseline) with exact permutation of the remission coefficient, adjustment for baseline inflammation score, Hodges-Lehmann and median differences, and weighting by the number of eligible site pairs; these archived alternative change models used at most three parameters; the added batch and pseudobulk models are described separately. Cell-level linear mixed models [35,36] with random intercepts for patient and biopsy (and a random pseudotime slope by biopsy for slope endpoints) tested the treatment-by-remission interaction with Satterthwaite degrees of freedom. Patient influence was assessed by leaving out each patient in turn.

### Exploratory batch diagnostics and donor pseudobulk analyses

We added the following analyses after peer review, before inspecting their new results. Batch diagnostics counted independent patients by outcome and sequencing batch, checked design rank and the residual variation in outcome after batch adjustment, and enumerated outcome-label permutations within each batch while preserving its remission count. The statistic was the remission coefficient from regression of patient-level post-minus-pre change on outcome and batch indicators. This conditional test requires exchangeability of patients within batch under the null; outcome was observational and was not randomized. The 14 program endpoints formed one Benjamini–Hochberg family for each specification. We examined the shared batches, each informative batch separately, and omission of individual patients or batches (S13 Table).

For transcriptome-wide analysis, we summed raw integer monocyte/macrophage counts within each patient and time point across the same eligible tissue sites, producing 30 pseudobulk libraries from 15 patients. Duplicate gene symbols, if any, were summed, with Ensembl-to-symbol mappings retained. We applied edgeR filterByExpr [37] using the four outcome-by-time groups, TMM library normalization, robust dispersion estimation and quasi-likelihood generalized linear models. Patient fixed effects accounted for paired observations; post-treatment and post-treatment-by-remission terms estimated the difference in change. Batch-adjusted models replaced the common post-treatment term with batch-specific post-treatment terms. Batch main effects were absorbed by patient effects because every patient belonged to one batch. We checked full design rank and retained residual degrees of freedom. Sensitivity models used the nine patients in batches containing both outcomes and all 26 available CD site pairs. Sites and cells were never counted as independent donors (S14 Table).

Seventeen explicitly curated enzyme or complex transcript modules resolved glycolysis, pyruvate entry, the TCA cycle, oxidative and nonoxidative pentose phosphate reactions, respiratory complexes I–IV, ATP synthase, fatty-acid synthesis and oxidation, cholesterol synthesis and transport, and lysosomal acidification and hydrolases. Membership was fixed in the analysis script before model fitting and is fully listed in S14 Table. These targeted modules do not constitute an exhaustive database enrichment screen. Using the same paired design, limma voom and fry [38] tested directional and mixed-direction module responses; all 34 tests were adjusted together within each model. Genes were adjusted across all expressed genes tested in that model. A separate limma voom gene analysis assessed agreement with edgeR. Sensitivity specifications were reported completely and were not selected by significance. Neither transcript modules nor model coefficients measure enzyme activity or metabolic flux.

### Cohort-selection sensitivity analyses

The selection rule excluded site pairs rather than whole patients: all 15 paired CD patients retained at least one eligible site. We re-estimated equal-site, within-patient program changes using thresholds of 1, 5, 10, 20, 30 and 50 cells at each time point, including descriptive low-cell estimates for all 26 pairs. The original global outcome-blind continuum and scoring definitions were held fixed, isolating the effect of site inclusion. Slopes required at least three cells and three distinct pseudotimes per biopsy; all actual tested-pair counts were retained. Each threshold was tested with and without batch adjustment and corrected across 14 endpoints. Low-cell estimates have greater sampling uncertainty and are sensitivity analyses.

We additionally varied unobserved reliable changes in excluded sites under a stated selection assumption: each excluded site was assigned its donor’s eligible-site mean change plus a common shift of −2, −1, 0, 1 or 2 pooled donor standard deviations. Each scenario was re-averaged within patient and batch-stratified. These values are hypothetical sensitivity parameters rather than measured effects or clinical imputations. Finite sensitivity ranges are conditional on their assumptions; the data do not provide assumption-free bounds. Inclusion weighting was not used because the cell-threshold rule is deterministic and cannot restore missing reliable site measurements (S15 Table).

### Positive controls

Three tiers of positive control were distinguished. Cell-identity controls tested whether independent monocyte and macrophage markers differed between S100A8/A9-high monocytes and C1Q-high IL1B-low macrophages within patients. Trajectory controls tested within-patient correlation between DPT and the independent maturation score. Outcome controls were 11 benchmarks fixed from the TAURUS publication before any were tested: the baseline C1Q-high IL1B-low macrophage proportion; the post/pre myeloid fraction of all cells; the within-remission change in S100A8/A9-high monocyte and C1Q-high IL1B-low macrophage proportions; the longitudinal remission-by-time change in *TNFRSF1B*, *CTSB*, *CTSS* and *TREM1* in S100A8/A9-high monocytes and *ETS2* in C1Q-high IL1B-high macrophages; and baseline *TNF* and *CXCL3* in TNF-high monocytes [2]. Each was tested at the patient level by pseudobulk averaging (at least 5 cells of the state per biopsy) and exact permutation, and the published mixed-effects association of single cells (MASC) models [39] were refitted with lme4 to locate any discrepancy. Four literature-based marker programs (inflammatory, resident maturation, TREM2/IL-10 regulatory and interferon response) were also tested.

### Detection limits

Empirical power was estimated with the actual exact test: observed patient residuals were resampled within outcome groups, a shift of d pooled standard deviations was added to the remission group (d from 0 to 3 in steps of 0.25; 2,000 simulations per step), and the 3,003-allocation test was applied. Family-level power resampled whole patients to preserve correlation among the 14 endpoints and applied Benjamini-Hochberg correction (1,000 simulations per step). Prospective sample sizes used a two-sample t test with 2:1 remission-to-nonremission allocation. These calculations describe design sensitivity and are not evidence for the absence of an effect.

### Myeloid recovery and selection

Total post-quality-control cells per biopsy, sequencing depth and median unique molecular identifiers per cell were taken from source S1 Table and agreed exactly with the atlas-wide cell table (987,743 cells). Myeloid and monocyte/macrophage fractions of all recovered cells, log post/pre ratios averaged within patient, and the published MASC compartment model were analysed. Eligible and excluded site pairs were compared descriptively.

### Independent datasets

We searched GEO, ArrayExpress, CELLxGENE, the Human Cell Atlas, the Broad Single Cell Portal, Zenodo and the literature for longitudinal intestinal single-cell datasets with anti-TNF exposure, patient identifiers and outcomes (S11 Table). Only a pediatric terminal-ileum study met the design criteria in part [16]: 6 treated patients had repeat biopsies (3 full and 3 partial responders, outcome at 2 years, infliximab or adalimumab), so the smallest attainable two-sided exact P for an outcome contrast is 0.10, and its public portal record describes only the diagnostic biopsies, with file access requiring an authenticated account. No dataset could therefore replicate the longitudinal outcome contrast. As a partial, cross-sectional check of continuum organization only, we analysed author-annotated monocytes and macrophages from colonic biopsies of 18 donors with CD, UC or no IBD (GSE214695) [9] using the same root rule, DPT parameters and scoring formula, and compared within-donor program gradients with those in TAURUS.

### Ulcerative colitis

UC was not analysed for outcome inference in this revision. Only 3 UC patients in remission met the cell threshold, so the minimum attainable two-sided exact P is 1/286 (0.0035), and a single extreme allocation determines significance. Individual patient values from the original exploratory occupancy analysis are provided descriptively in S12 Table.

### Software, reproducibility and use of artificial intelligence tools

Analyses used Python 3.12 (AnnData 0.13.2, Scanpy 1.12.3, NumPy 2.3.5, SciPy 1.18.0, pandas 3.0.5, statsmodels 0.14.6, scikit-learn 1.7.2), a separate environment for Palantir 1.4.1, and R 4.6.1 with Bioconductor 3.23 (slingshot 2.20.0, UCell 2.16.0, SingleCellExperiment 1.34.0, bluster 1.22.0, rhdf5 2.56.1, lme4 2.0.6 and lmerTest 3.2.1). Random procedures used fixed seeds. Scripts, environment files, session information, logs, checksums and all patient-level intermediate tables are provided in the reproducibility archive; the revision reproducibility package is identified by DOI 10.5281/zenodo.23397636 (version 1.0.0). The large public source matrix is available from the corrected upstream TAURUS release; the revision archive provides its retrieval helper and checksum along with small input files and complete derived results. OpenAI ChatGPT and Codex assisted with code, drafting and formatting of the original submission and first revision, and Anthropic Claude (Claude Code) assisted with code. No data, patient observations or results were generated by these tools outside the documented, executed scripts. The author wrote, reviewed and approved the revised manuscript, confirmed the author declarations, and takes responsibility for the study design, analysis oversight, validation, interpretation and final content. AI assistance is disclosed here.

### Ethics statement

This secondary analysis used only publicly available, de-identified data and involved no recruitment, intervention or access to identifiable information. Ethical approval and informed consent for the original sample collection are reported by Thomas et al. [2]. No new institutional review board approval was required for this reanalysis.

## Results

### Cohort, selection and myeloid composition

Of the 26 CD site pairs, 19 from 15 patients met the 30-cell threshold: 13 pairs from 10 remission patients and 6 pairs from 5 nonremission patients (Fig 1A; Table 1). All 15 patients retained at least one pair. The 7 excluded pairs were all colonic, 6 of 7 were from remission patients, and most had very few post-treatment monocytes or macrophages (median 13 cells), so exclusion was related to outcome. Twelve of 19 eligible pairs, including 5 of 6 nonremission pairs, were from the terminal ileum. Nonremission patients had longer disease duration and ileal disease location more often, and eligible nonremission pairs came only from sequencing batches 1 and 3 (Table 1).

**Fig 1:**
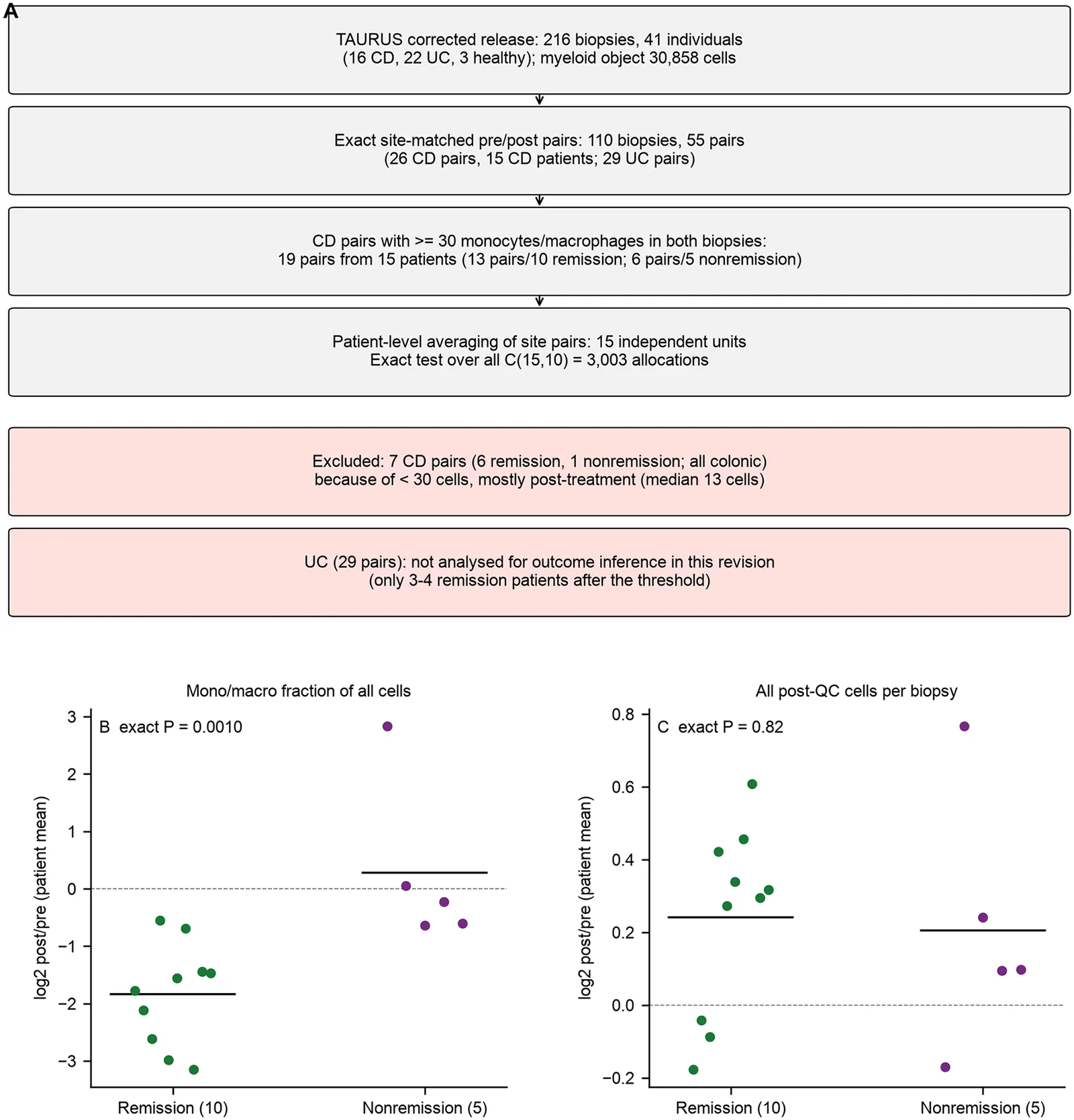
Cohort selection and outcome-associated change in myeloid composition. (A) Selection of the Crohn’s disease (CD) cohort from the corrected TAURUS release. (B) Patient-level log2 post/pre ratio of the monocyte/macrophage fraction of all post-quality-control cells per biopsy (all 26 CD site pairs, averaged within patient). (C) The same for the total number of post-quality-control cells per biopsy. Horizontal bars are group means. P values are two-sided exact permutation tests over 3,003 allocations. Panel B is a compositional measure within sequenced biopsies, not absolute tissue density.

Eligible remission biopsies contained 2,056 monocytes/macrophages before and 814 after treatment, compared with 1,037 and 2,194 in nonremission. Total recovered cells per biopsy did not differ by outcome (patient geometric mean post/pre ratio 1.18 versus 1.15; exact P = 0.82), and neither did sequencing depth nor cells loaded (S10 Table). Instead, the monocyte/macrophage fraction of all recovered cells fell in remission (post/pre 0.28 versus 1.22; P = 0.0010; all leave-one-patient-out P ≤ 0.009; Fig 1B and 1C). Refitting the published MASC compartment model reproduced this contraction (odds ratio after treatment 0.39 in remission and 0.98 in nonremission; interaction P = 0.008). The previously reported imbalance in recovered myeloid cells therefore reflects a change in myeloid composition within sequenced biopsies rather than a difference in overall cell recovery, although these data cannot measure absolute tissue cell density.

### Continuum and trajectory topology

The reproduced DPT ordered the released states from S100A8/A9-high monocytes (median 0.212) through TNF-high IL6-positive monocytes (0.373) and C1Q-high IL1B-high macrophages (0.541) to C1Q-high IL1B-low macrophages (0.652) (Fig 2A and 2B). DPT correlated with the independent maturation score (Spearman rho 0.75; S3 Fig), and within-patient correlations were positive in all 39 patients with at least 50 cells (median 0.72).

**Fig 2:**
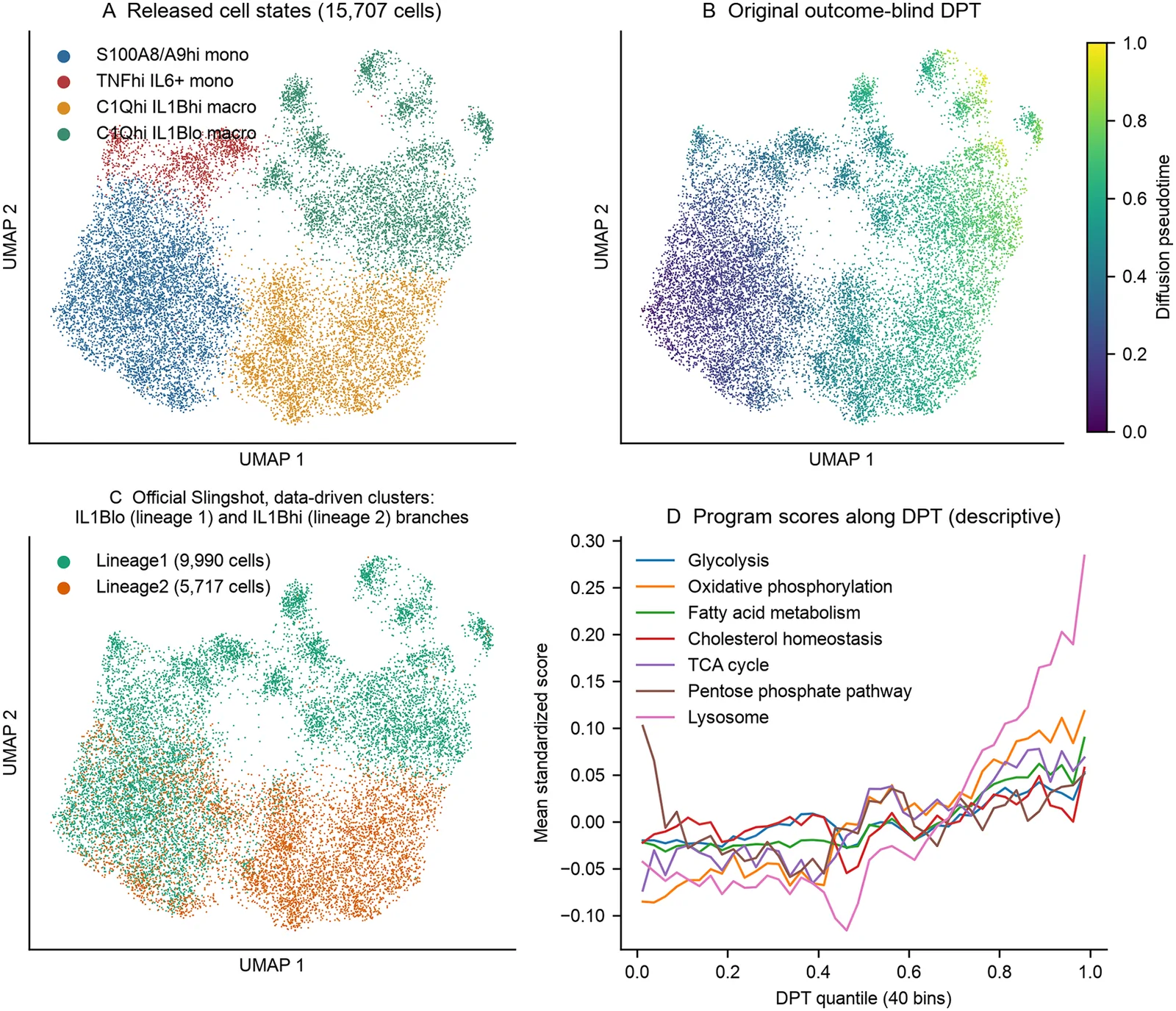
Outcome-blind monocyte-to-macrophage continuum and branch structure. (A) UMAP of 15,707 monocytes and macrophages coloured by released cell state. (B) Original diffusion pseudotime (DPT), rooted by a prespecified monocyte-minus-macrophage marker contrast without treatment or outcome labels. (C) Official Slingshot with annotation-free Louvain clusters on the released Harmony coordinates infers lineages ending in C1Q-high IL1B-low and C1Q-high IL1B-high macrophages; cells are shown by their largest curve weight, and monocytes are shared. (D) Mean standardized program scores in 40 DPT quantile bins. Curves are descriptive, pool cells across patients and do not measure metabolic flux.

Official Slingshot with the four released states as clusters inferred one unbranched lineage in all four specifications. With only four clusters the minimum spanning tree is necessarily a path, and this lineage placed IL1B-high macrophages after IL1B-low macrophages, against the independent maturation markers (rho 0.32). With annotation-free clusters, Slingshot separated the macrophages into branches: Louvain clusters gave two lineages from shared monocytes, one ending in C1Q-high IL1B-low macrophages (11,535 cells with weight ≥0.5) and one ending in C1Q-high IL1B-high macrophages (9,497 cells) (Fig 2C), and k-means clusters gave three lineages, including a monocyte-retained arm (S1 Fig; S3 Table; S4 Fig). Within each data-driven lineage, Slingshot pseudotime agreed closely with DPT (rho 0.96 and 0.97). Palantir pseudotime correlated with DPT at rho 0.98 but identified terminal states only in the IL1B-low region, and the metabolic-gene-free continuum correlated with DPT at rho 0.85 with the same state order (S2 Fig). The data are therefore consistent with a monocyte-to-resident-macrophage axis, while the position of IL1B-high macrophages, as a branch or an intermediate state, depends on the method.

### Positive controls

Independent markers differed between monocytes and macrophages within patients in the expected direction for 10 of 11 genes (the exception was *LYZ*; S5 Table). For the published outcome benchmarks, refitting MASC to the published population reproduced the baseline C1Q-high IL1B-low macrophage association exactly (odds ratio for nonremission 0.459; P = 0.0016), as well as the published longitudinal state-proportion results. The published population comprised all 26 paired pretreatment biopsies, not inflamed biopsies only; restricting to inflamed biopsies weakened the association (P = 0.074).

At the patient level, all 11 benchmarks reproduced the published direction (Fig 3; Table 2). The baseline C1Q-high IL1B-low proportion was higher in later remitters (0.240 versus 0.119; difference 0.121, 95% CI 0.069 to 0.174; exact P = 0.0027; studentized P = 0.0003; q = 0.004). The other two-group benchmarks had absolute Hedges g of 0.65 to 1.63, and 6 of the 11 benchmarks had robust P < 0.05, but after heteroskedasticity-robust testing and correction across the 11 benchmarks only the baseline macrophage benchmark remained significant. The previous revision’s non-significant result for this benchmark (P = 0.154) arose from restricting to the 19 eligible pretreatment biopsies and using monocytes and macrophages as the denominator; the direction was consistent across all 12 specifications examined (S5 Table). Because these benchmarks were discovered in the same data, their reproduction calibrates the patient-level pipeline but is not independent replication. Four literature marker programs showed no remission-specific change (S5 Table).

**Fig 3:**
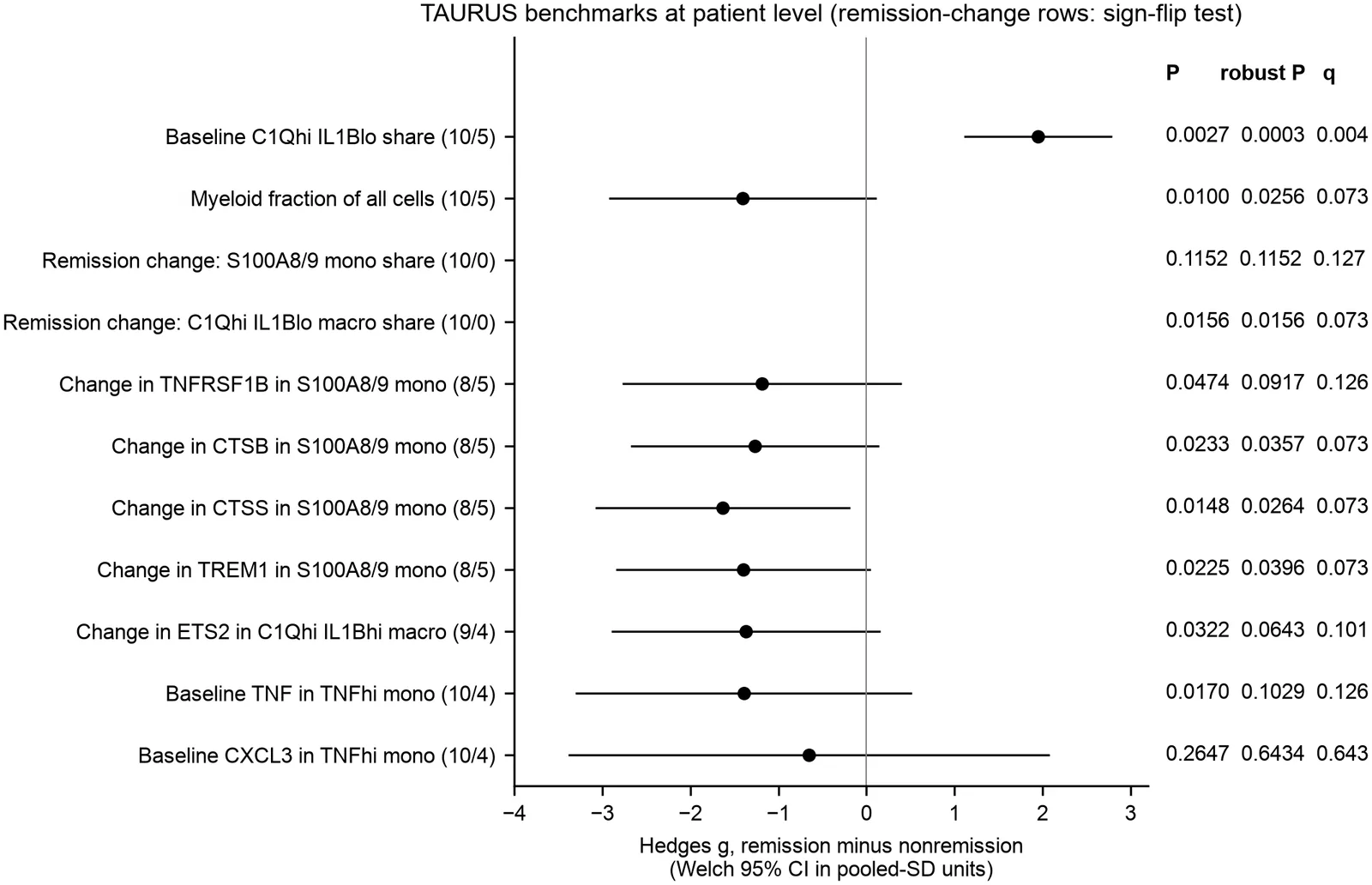
Published outcome-associated myeloid benchmarks reproduced with patient-level inference. Eleven benchmarks were fixed from the TAURUS publication before testing. Points are Hedges g with Welch 95% confidence intervals in pooled-SD units (patients per group in parentheses). Columns give the exact permutation P, the heteroskedasticity-robust P (studentized exact permutation; exact sign-flip for within-remission rows) and Benjamini-Hochberg q across the 11 benchmarks using the robust P. These reproductions use the data in which the benchmarks were discovered and are not independent replication.

**Table 2:** Published TAURUS myeloid benchmarks tested at the patient level.

Benchmark	Patients (R/NR)	Effect (95% CI)	Exact P	Robust P	Adjusted P	Published direction
Baseline C1Q-high IL1B-low macrophage proportion	10/5	0.121 (0.069 to 0.174)	0.0027	0.0003	0.004	Yes
Post/pre myeloid fraction of all cells	10/5	−0.829 (−1.725 to 0.067)	0.0100	0.0256	0.073	Yes
Change in remission: S100A8/A9-high monocytes proportion	10/0	−0.068 (−0.136 to 0.007)	0.1152	0.1152	0.127	Yes
Change in remission: IL1B-low macrophages proportion	10/0	−0.098 (−0.155 to −0.039)	0.0156	0.0156	0.073	Yes
Change in *TNFRSF1B* in S100A8/A9-high monocytes	8/5	−0.416 (−0.972 to 0.140)	0.0474	0.0917	0.126	Yes
Change in *CTSB* in S100A8/A9-high monocytes	8/5	−0.472 (−0.996 to 0.052)	0.0233	0.0357	0.073	Yes
Change in *CTSS* in S100A8/A9-high monocytes	8/5	−0.649 (−1.223 to −0.075)	0.0148	0.0264	0.073	Yes
Change in *TREM1* in S100A8/A9-high monocytes	8/5	−0.379 (−0.771 to 0.014)	0.0225	0.0396	0.073	Yes
Change in *ETS2* in IL1B-high macrophages	9/4	−0.267 (−0.564 to 0.031)	0.0322	0.0643	0.101	Yes
Baseline *TNF* in TNF-high monocytes	10/4	−0.834 (−1.975 to 0.307)	0.0170	0.1029	0.126	Yes
Baseline *CXCL3* in TNF-high monocytes	10/4	−0.322 (−1.672 to 1.028)	0.2647	0.6434	0.643	Yes

Benchmarks were fixed from Thomas et al. before testing. Proportions are of mononuclear phagocytes (monocytes, macrophages and dendritic cells). Effects are remission minus nonremission (two-group rows; Welch 95% CI) or mean change in remission (sign-flip rows; bootstrap 95% CI). Robust P is the studentized exact permutation P for two-group rows and the exact sign-flip P otherwise; adjusted P is Benjamini-Hochberg across the 11 benchmarks using the robust P. Gene expression is mean log-normalized expression in the stated state (at least 5 cells per biopsy). These are reproductions in the discovery data, not independent replication.

### Primary metabolic contrasts

No program level or slope change differed between remission and nonremission (Table 3; Fig 4). The smallest exact P among the 14 primary tests was 0.397 (fatty acid metabolism level), all Benjamini-Hochberg adjusted P values were 0.987, the smallest studentized P was 0.429, and the exact family-wise maximum-statistic P was 0.980. Standardized differences ranged from −0.33 to 0.45 SD. For example, the glycolysis level difference was −0.005 (95% CI −0.052 to 0.042), the lysosome level difference 0.042 (−0.097 to 0.181) and the OXPHOS slope difference −0.112 (−0.455 to 0.231). Scores are in units of per-gene standardized expression and slopes in score units per unit of DPT.

**Table 3:** Primary patient-level metabolic-program contrasts in Crohn’s disease.

Program	Metric	ΔR	ΔNR	Difference (95% CI)	g	Exact P	Stud. P	q
Glycolysis	Level	0.002	0.007	−0.005 (−0.052 to 0.042)	−0.10	0.839	0.816	0.987
Glycolysis	DPT slope	0.023	0.042	−0.019 (−0.228 to 0.191)	−0.10	0.854	0.849	0.987
Oxidative phosphorylation	Level	−0.065	−0.064	−0.001 (−0.179 to 0.178)	−0.01	0.987	0.988	0.987
Oxidative phosphorylation	DPT slope	−0.045	0.067	−0.112 (−0.455 to 0.231)	−0.33	0.534	0.486	0.987
Fatty acid metabolism	Level	0.004	−0.021	0.025 (−0.048 to 0.098)	0.45	0.397	0.429	0.987
Fatty acid metabolism	DPT slope	−0.004	0.053	−0.057 (−0.243 to 0.129)	−0.30	0.562	0.511	0.987
Cholesterol homeostasis	Level	0.006	0.004	0.002 (−0.061 to 0.065)	0.03	0.958	0.949	0.987
Cholesterol homeostasis	DPT slope	0.077	0.094	−0.017 (−0.372 to 0.339)	−0.05	0.927	0.916	0.987
TCA cycle	Level	−0.021	−0.056	0.035 (−0.141 to 0.211)	0.34	0.518	0.621	0.987
TCA cycle	DPT slope	−0.089	0.009	−0.098 (−0.449 to 0.253)	−0.27	0.610	0.552	0.987
Pentose phosphate pathway	Level	−0.066	−0.068	0.002 (−0.117 to 0.122)	0.03	0.948	0.960	0.987
Pentose phosphate pathway	DPT slope	−0.111	−0.089	−0.022 (−0.377 to 0.332)	−0.09	0.870	0.880	0.987
Lysosome	Level	0.031	−0.011	0.042 (−0.097 to 0.181)	0.35	0.510	0.512	0.987
Lysosome	DPT slope	0.057	−0.025	0.082 (−0.239 to 0.402)	0.25	0.664	0.636	0.987

Values are patient-level post-minus-pre changes (10 remission and 5 nonremission patients; multiple site pairs averaged within patient). Levels are in units of per-gene standardized log-normalized expression; slopes are in score units per unit of diffusion pseudotime (DPT). Exact P values enumerate all 3,003 outcome allocations; studentized P uses the Welch statistic. Adjusted P is Benjamini-Hochberg across the 14 tests. The originally reported stratified bootstrap intervals are given in S7 Table. ΔR and ΔNR are group mean changes in remission and nonremission; g is Hedges g; Stud. P is the studentized exact P; q is the Benjamini–Hochberg adjusted P.

**Fig 4:**
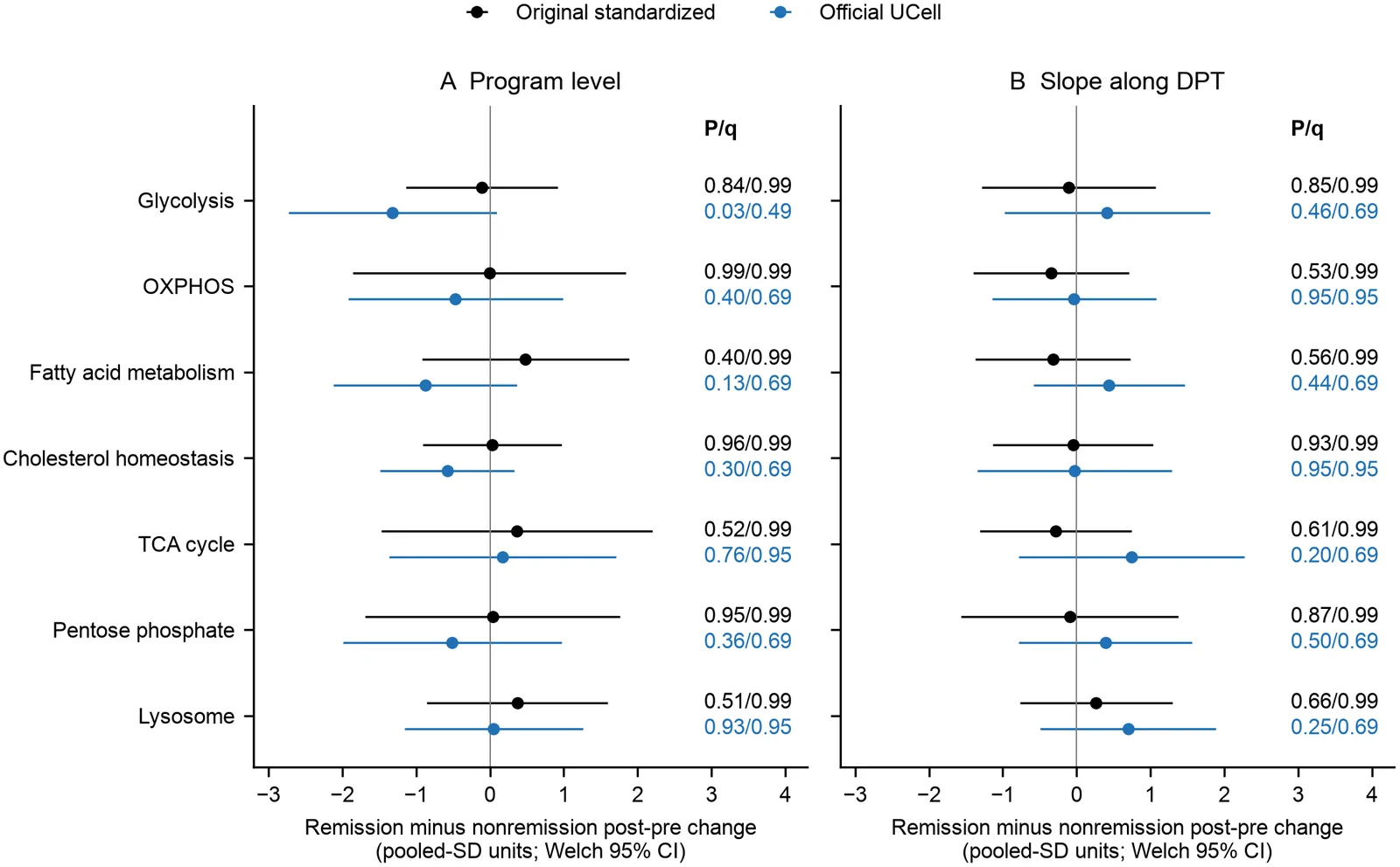
Primary patient-level metabolic contrasts with original and official UCell scoring. Remission (n = 10) minus nonremission (n = 5) difference in patient-level post-minus-pre change for (A) program level and (B) slope along diffusion pseudotime, in pooled-SD units with Welch 95% confidence intervals. Numbers give the exact permutation P and the Benjamini-Hochberg q within each 14-test scoring family.

With official UCell scoring, no contrast was significant after correction (minimum adjusted P = 0.49; Fig 4). The glycolysis level contrast had a nominal P of 0.035 at the default rank cutoff but 0.109 and 0.568 at cutoffs of 800 and 3,000, so it was not robust. UCell and original scores correlated at the cell level (rho 0.67 to 0.91), and the direction of the remission contrast agreed for 4 of 7 program levels, all of which were small relative to their uncertainty (S5 Fig). The archived rank score used in the original submission, which ranked genes only within the metabolic gene universe, was negatively correlated with the original glycolysis score (rho −0.38) and is therefore not an adequate second method (S4 Table).

### Change during treatment in both outcome groups

Across all 15 patients, the pentose phosphate pathway level decreased after treatment (mean change −0.067; sign-flip P = 0.0013; q = 0.018), and OXPHOS decreased nominally (−0.064; P = 0.016; q = 0.114); the corresponding UCell decreases were also present (q = 0.025 and 0.040). Decomposition showed these changes to be within states rather than compositional (S6 Fig). Over the same interval the mitochondrial read percentage rose by 2.2 percentage points (P = 0.0017), and no gene in the 31-enzyme panel changed significantly after correction (minimum q = 0.12). Ambient epithelial transcripts in monocyte/macrophage droplets also increased after treatment in both groups (P = 0.0005; outcome difference P = 0.83). After adjustment for mitochondrial read percentage, the pentose phosphate decrease persisted (q = 0.048) whereas the OXPHOS decrease did not (q = 0.30). Without an untreated comparison, and with concurrent changes in stress-sensitive and ambient technical measures, these within-patient changes cannot be attributed to adalimumab or to altered metabolism.

### Robustness to trajectory, scoring, composition, site and model

Branch-specific analyses along official Slingshot lineages found 3 nominal P values below 0.05 among 70 tests in 5 lineage families, about the number expected by chance, with no adjusted P below 0.47; the k-means monocyte-retained arm could not be tested because only 2 remission patients had sufficient cells (Table 4; S3 Table). Palantir and the metabolic-gene-free continuum gave minimum adjusted P values of 0.97 and 0.98. Within-state analyses, the composition decomposition, restriction to common pseudotime support, Theil-Sen and Spearman summaries and the binned functional contrast were all null after correction (minimum adjusted P 0.48; S9 Table). Terminal-ileum-only analysis (7 remission and 5 nonremission patients) gave a minimum adjusted P of 0.82, and a colon-only analysis was not possible because only one nonremission patient contributed colonic pairs. Alternative patient-level models, including baseline-adjusted analysis of covariance, gave no adjusted P below 0.92, and cell-level mixed models gave none below 0.45 (Satterthwaite degrees of freedom 18–67, reflecting replication at the biopsy and patient levels; S8 Table). Because eligible nonremission pairs came only from sequencing batches 1 and 3, we repeated the primary analysis in patients from these batches (4 remission and 5 nonremission patients). Three slope contrasts (OXPHOS, fatty acid metabolism and cholesterol homeostasis) had nominal P values of 0.016 to 0.048, with a more negative slope change in remission, but none remained significant after correction (minimum adjusted P 0.22). Across all 481 exploratory remission-contrast tests, 10 (2.1%) had nominal P < 0.05, fewer than the 24 expected by chance.

**Table 4:** Sensitivity analyses of the remission contrast.

Analysis	Patients (R/NR)	Tests	Minimum P	Minimum adjusted P
*Trajectory method*				
Official Slingshot, annotated states (1 lineage)	10/5	14	0.048	0.68
Official Slingshot, Louvain lineage to IL1B-low macrophages	7/5	14	0.037	0.51
Official Slingshot, Louvain lineage to IL1B-high macrophages	9/5	14	0.235	0.86
Official Slingshot, k-means lineage 1	10/5	14	0.033	0.47
Official Slingshot, k-means lineage 2	6/5	14	0.052	0.73
Official Slingshot, k-means monocyte-retained lineage	2/5	0		not tested
Palantir pseudotime (slopes)	10/5	7	0.453	0.97
Metabolic-gene-free DPT (slopes)	10/5	7	0.334	0.98
*Scoring method*				
Official UCell, rank cutoff 1,500	10/5	14	0.035	0.49
Official UCell, rank cutoff 800	10/5	14	0.109	0.84
Official UCell, rank cutoff 3,000	10/5	14	0.291	0.94
Official UCell, Slingshot slopes	10/5	7	0.022	0.16
*Composition, support and shape*				
Within C1Q-high IL1B-low macrophages	8/5	7	0.069	0.48
Within C1Q-high IL1B-high macrophages	8/4	7	0.202	0.98
Within S100A8/A9-high monocytes	3/5	7	0.250	0.95
Decomposition: within-state component	10/5	7	0.428	0.95
Decomposition: composition component	10/5	7	0.259	0.91
Common pseudotime support	10/5	14	0.265	0.98
Theil-Sen slopes	10/5	7	0.320	0.92
Binned functional contrast	10/5	7	0.590	0.94
Terminal ileum only	7/5	14	0.307	0.82
*Model*				
Studentized exact permutation	10/5	14	0.429	0.99
Baseline-adjusted ANCOVA (HC3)	10/5	14	0.295	0.93
Adjusted for baseline inflammation (HC3)	10/5	14	0.154	0.92
Hodges-Lehmann shift	10/5	14	0.536	0.98
Cell-level mixed model, original scores	10/5	14	0.409	0.99
Cell-level mixed model, UCell scores	10/5	14	0.057	0.45
Exact family-wise maximum statistic	10/5	14	0.397	0.98
*Subset*				
Batch-matched subset (batches 1 and 3)	4/5	14	0.016	0.22

Each family was adjusted by Benjamini-Hochberg within the family (7 or 14 tests); the maximum-statistic row gives the smallest family-wise adjusted P. R, remission; NR, nonremission; DPT, diffusion pseudotime; ANCOVA, analysis of covariance; HC3, heteroskedasticity-consistent standard errors. Full results are in S3, S4, S8 and S9 Tables.

### Detection limits, interval behavior and patient influence

With the observed residual distributions, the exact test reached 80% power for a single endpoint at shifts of 1.38 to 1.57 pooled SD (90% power at 1.52 to 1.80 SD), and at 2.1 to 2.4 SD with Bonferroni correction for 14 tests (Fig 5A and 5B; S7 Table). With Benjamini-Hochberg correction, a shift confined to one endpoint required about 2.2 SD for 80% power, whereas a shift common to all 14 endpoints was detected with 80% probability at about 1.3 SD. The largest observed absolute standardized difference was 0.45 SD. A standard two-sample t calculation gave a comparable 80%-power effect of 1.66 SD for 10 and 5 patients. Leaving out each patient changed the sign of the point estimate in 37 of 210 recalculations across 9 endpoints (up to 7 of 15 for cholesterol homeostasis level), but no recalculated P fell below 0.162; patients CD12 and CD11, both in nonremission, were the most influential (Fig 5C; S6 Table).

**Fig 5:**
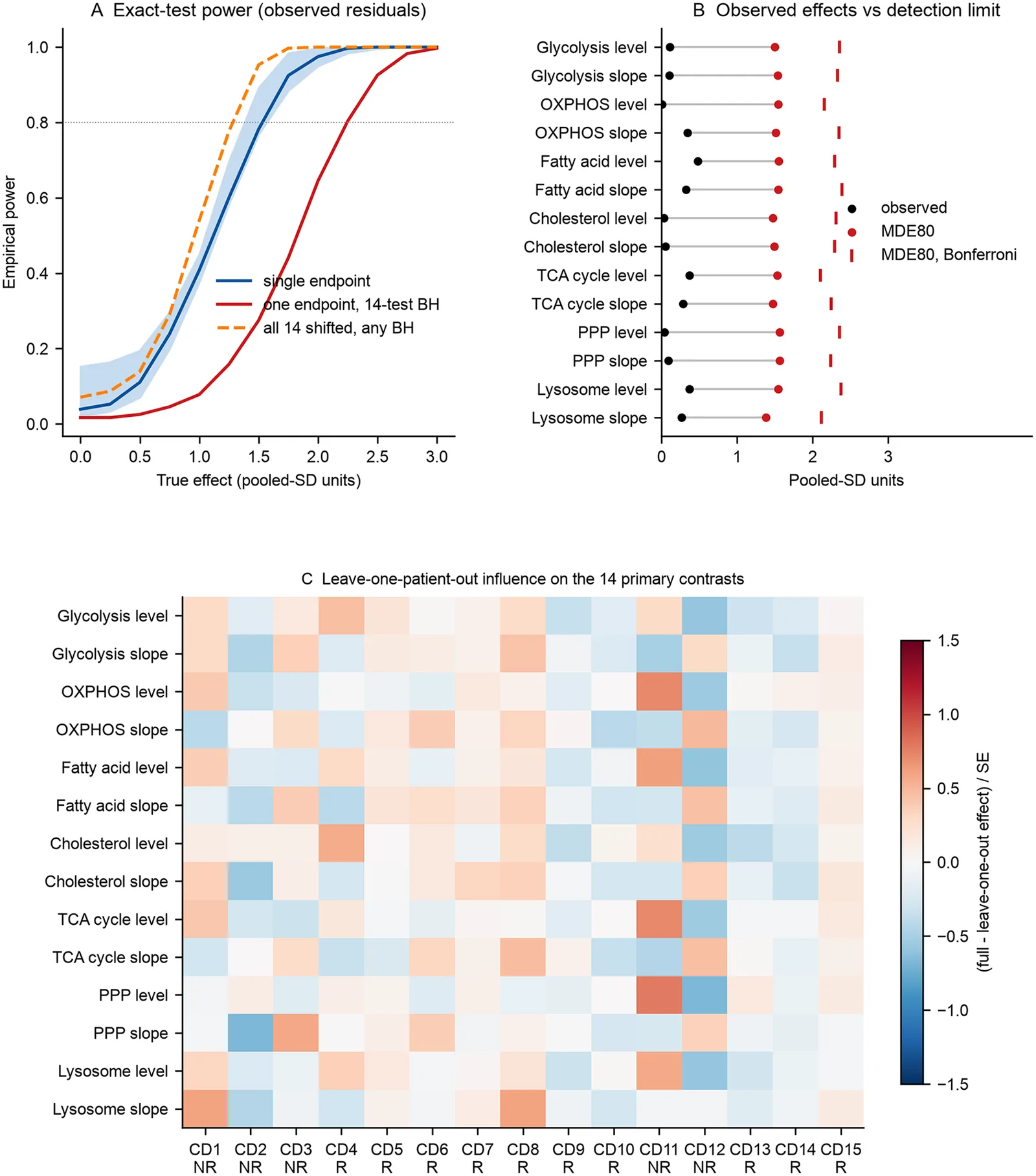
Detection limits and patient influence. (A) Empirical power of the exact permutation test when a shift is added to resampled observed patient residuals, for single endpoints (median and range across 14 endpoints) and jointly across the 14 correlated endpoints with Benjamini-Hochberg correction. (B) Observed absolute contrasts and minimum detectable effects (MDE) at 80% power, unadjusted and with Bonferroni correction. (C) Change in each primary contrast, in standard-error units, when each patient is omitted (R, remission; NR, nonremission). Post hoc power describes design sensitivity and does not support a conclusion of no effect.

### Partial external check of continuum organization

In GSE214695 (2,009 author-annotated monocytes and macrophages from 18 donors; 15 donors with at least 30 cells), program gradients along the marker-based monocyte-to-macrophage axis had the same median sign as in TAURUS for 6 of 7 programs, including OXPHOS and lysosome in 14 and 14 of 15 donors, whereas the pentose phosphate gradient was near zero in both datasets (S8 Fig). A DPT recomputed in this smaller dataset without batch correction ordered macrophage subsets differently and did not reproduce the gradients. This cross-sectional comparison supports, conditionally on the ordering, the general organization of program scores along the monocyte-to-macrophage axis; it is not a replication of the treatment-outcome contrast.

### Batch confounding and transcriptome-wide donor analyses

Batch 1 contained 1 remission and 3 nonremission patients, batch 2 contained 4 remission patients, batch 3 contained 3 remission and 2 nonremission patients, and batch 4 contained 2 remission patients. Batch explained 41.5% of the patient-level variation in outcome. The patient-change regression had full rank (5 columns), but within-batch comparisons were informed by only nine patients in batches 1 and 3. There were 40 conditional label allocations. The smallest batch-stratified P value was 0.025 and the smallest adjusted P across 14 endpoints was 0.350; the shared-batch analysis gave the same coefficient and conditional P values. No within-batch result survived correction. Patient and batch omission analyses are reported in full in S13 Table. Time-invariant additive batch effects cancel in paired changes, whereas batch-dependent changes remain a possible confounder.

The primary pseudobulk analysis tested 7092 expressed genes. No gene survived Benjamini–Hochberg correction in the unadjusted model (minimum adjusted P 0.298) or in the model allowing batch-specific post-treatment changes (minimum adjusted P 0.894). The adjusted model had rank 20 of 20 and 10 residual degrees of freedom. Models restricted to shared batches (7739 genes; 6 residual degrees of freedom) and including all 26 site pairs (7021 genes; 10 residual degrees of freedom) also yielded no corrected gene findings. The independent limma voom model detected no gene at adjusted P < 0.05 (minimum adjusted P 0.421); log-fold-change estimates correlated with edgeR estimates (Spearman ρ = 0.965). These analyses extend resolution beyond the previous 31-gene panel but remain limited by the small number of independent patients (Fig 6; Table 5; S14 Table).

**Fig 6:**
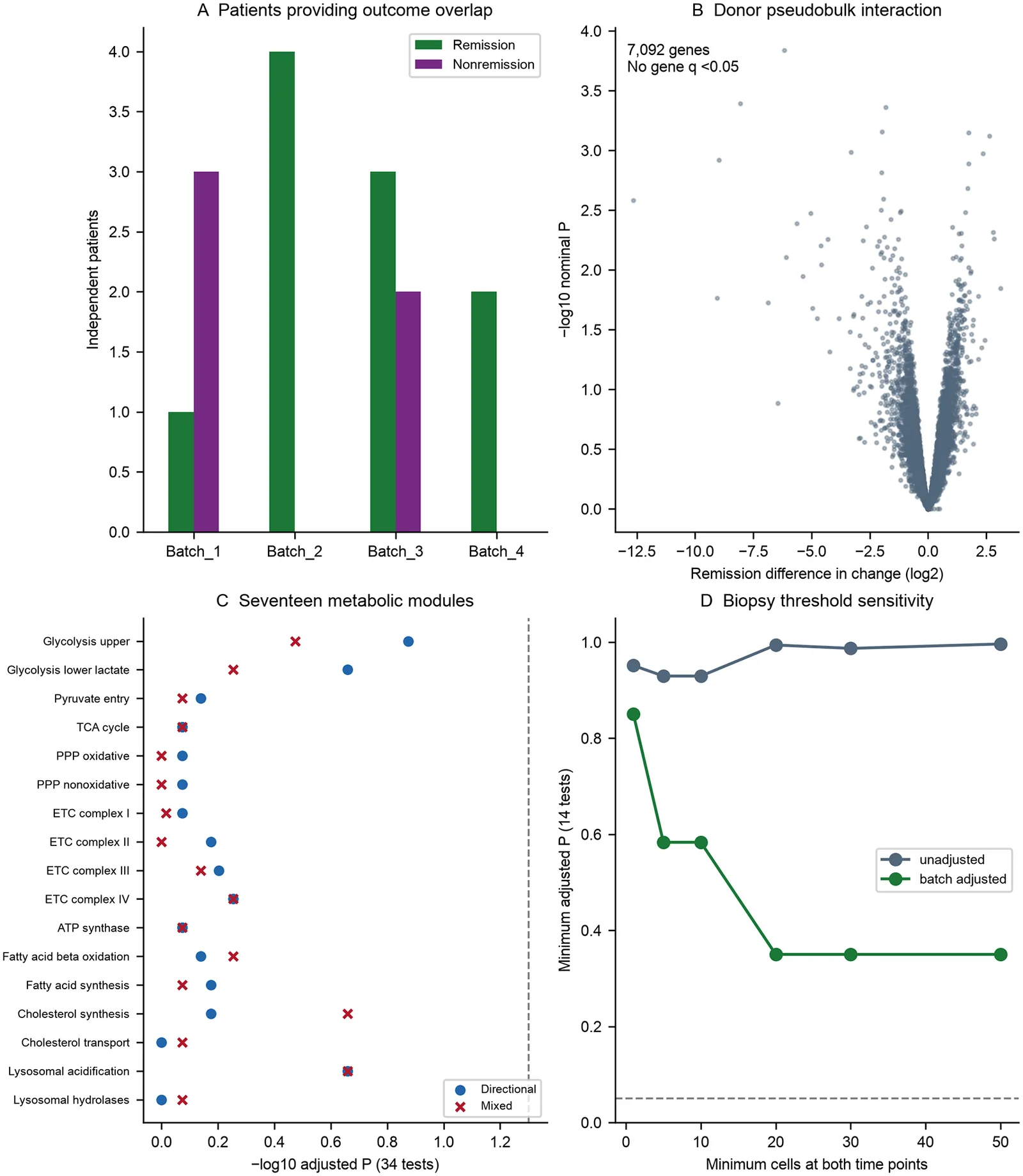
Targeted analyses of batch structure, donor pseudobulk transcription and biopsy selection. (A) Independent patients by sequencing batch and outcome. (B) Transcriptome-wide edgeR quasi-likelihood interaction tests in the primary batch-adjusted model (7,092 genes); all adjusted P values exceed 0.05. (C) Directional and mixed-test adjusted P values for all 17 curated metabolic transcript modules; Benjamini–Hochberg correction across 34 tests. The vertical line marks adjusted P = 0.05. (D) Minimum adjusted P among 14 program contrasts across cell thresholds, with and without batch adjustment. These minima summarize complete test families; no threshold was selected as confirmatory. Full results are in S13–S15 Tables.

**Table 5:** Transcriptome-wide donor pseudobulk and metabolic module model summaries.

Specification	Donors	Genes	Residual df	Min gene q	Min module q
Primary paired	15	7092	13	0.298	0.206
Primary batch-adjusted	15	7092	10	0.894	0.134
Shared batches	9	7739	6	0.959	0.280
All 26 pairs, batch-adjusted	15	7021	10	0.778	0.153

All coefficients test the difference in post-minus-pre change between remission and nonremission. Patient effects account for pairing. Batch-adjusted models include batch-specific post-treatment terms. Genes are corrected across all tested genes; module q values jointly correct 17 directional and 17 mixed tests. No gene or module had q < 0.05. No cell, site or pseudobulk library was treated as an independent patient.

None of the 17 metabolic transcript modules was significant after joint correction of directional and mixed tests in any model. In the primary batch-adjusted model, upper glycolysis had the smallest directional P value (0.00393), but its adjusted P across all 34 module tests was 0.134. This nominal observation is exploratory and does not establish a subpathway effect. All module memberships, expressed-gene counts and both test types are provided in S14 Table and S10 Fig.

### Sensitivity to biopsy-pair selection

Seven of 26 CD site pairs failed the primary 30-cell rule. All seven were colonic and six belonged to remission patients; no paired CD patient was excluded completely. Thresholds of 1, 5, 10, 20, 30 and 50 retained 26, 25, 25, 20, 19 and 14 pairs, respectively, from 15, 15, 15, 15, 15 and 12 patients. None of the 14 contrasts survived correction at any threshold, with or without batch adjustment. The all-pair batch-adjusted minimum adjusted P was 0.850 compared with 0.350 at the primary threshold. The hypothetical excluded-site scenarios from −2 to +2 SD yielded no corrected contrast (smallest adjusted P 0.117). These checks show sensitivity over the evaluated rules and assumptions; they do not remove site-selection bias or recover reliable measurements from sparsely sampled sites (Fig 6; S15 Table).

## Discussion

In this patient-level reanalysis of 15 patients with CD, we found no remission-specific change in seven metabolic transcriptional programs of intestinal monocytes and macrophages after adalimumab, either in overall program level or along an outcome-blind continuum. This held with the original and official UCell scoring, with diffusion pseudotime, official Slingshot lineages, Palantir and a continuum built without metabolic genes, and in within-state, composition-decomposed, common-support, site-restricted and mixed-model analyses. The same pipeline reproduced published outcome-associated myeloid findings in their published direction, robustly for the baseline C1Q-high IL1B-low macrophage proportion, which shows that the patient-level design can detect large outcome-associated differences in these data.

The primary tests did not detect remission-associated differences in program change. The simulated 80%-power thresholds of approximately 1.4–1.6 SD describe repeated-sampling sensitivity under the specified residual model; they are not confidence bounds and do not establish that effects above or below a threshold are absent or unlikely. Moderate and larger effects may remain compatible with endpoint-specific uncertainty. No independently justified equivalence margin was available. Leave-one-out analyses showed unstable signs for several estimates. Published benchmarks detected in the same data demonstrate some analytical sensitivity but cannot establish sensitivity to every metabolic contrast. The prospective sample-size calculations in S7 Table are planning estimates rather than evidence about effects in the current cohort.

The trajectory analyses qualify the original continuum. A single diffusion pseudotime is a reasonable summary of the monocyte-to-resident-macrophage axis: it agreed with independent maturation markers, with Palantir and with a metabolic-gene-free embedding. However, annotation-free Slingshot placed IL1B-high and IL1B-low macrophages on separate branches, whereas Slingshot on the four annotated states and Palantir did not, so the topology of IL1B-high macrophages cannot be settled from these data. This uncertainty did not change the metabolic conclusions, because branch-specific tests were also null, but pseudotime slopes are summaries of an expression ordering and not rates of differentiation.

Myeloid composition changed with outcome. The monocyte/macrophage fraction of all recovered cells fell after treatment in remission but not in nonremission, while no outcome difference was detected in total cell recovery or sequencing depth. This agrees with the myeloid contraction reported by the source study and is expected from the definition of remission, which requires endoscopic or histological improvement and therefore resolution of inflammatory infiltrates. Composition change also affected selection: the excluded pairs were all colonic, and six of seven came from remission patients; many had few post-treatment myeloid cells. Program levels averaged over all monocytes and macrophages combine changes within states with changes in state mixture, which is why we decomposed them; the within-state and composition components were both null for the remission contrast.

The decreases in pentose phosphate pathway and OXPHOS scores observed in both outcome groups should not be read as treatment effects. They lack an untreated comparison, they occurred together with a rise in mitochondrial read percentage, and mitochondrial, ribosomal and metabolic transcripts are sensitive to dissociation and storage. More generally, broad RNA program scores partly track cell identity, overlap with one another and do not measure enzyme activity or flux. Our results therefore do not contradict experimental evidence that glycolytic activation of CD14-positive macrophages supports inflammation in CD [12] or that macrophage IL-10 signaling contributes to anti-TNF efficacy [13]; the present cohort did not yield a corrected remission contrast in the tested transcriptional summaries.

This study has important limitations. It is a secondary analysis of one cohort with 15 independent patients and only 5 in nonremission, and no comparable longitudinal cohort was available for replication. Remission was a composite clinical, endoscopic and histological outcome assessed after a variable interval, and dosing, concomitant medication and patient-level timing were not available for adjustment. Nonremission pairs were concentrated in the terminal ileum and in two sequencing batches, so site and batch are partly confounded with outcome, although pre- and post-treatment biopsies always shared a batch; only nine patients provided within-batch outcome overlap. Adjusted models were estimable under common-effect assumptions, but they cannot exclude batch-dependent, site-dependent or subset-specific effects. Patients were classified by an outcome that itself reflects inflammation, and baseline differences permit regression toward the mean, which the baseline-adjusted analysis addresses only partly. Program scores depend on cell identity and composition, the positive controls are reproductions in the discovery data rather than independent validation, and outcome-related selection removed seven colonic site pairs, six from remission patients. The original analysis plan was archived before outcome modeling, but its timing cannot be verified independently, and all analyses added after review are exploratory. RNA velocity could not be estimated because spliced and unspliced counts are not publicly released, and transcriptomic data cannot establish metabolic flux or causality.

The targeted analyses increase transcriptional resolution and make the remaining uncertainty more explicit. Gene-level and module-level testing yielded no corrected finding, including with batch-specific time effects. Upper glycolysis was nominally associated with outcome but did not survive the declared 34-test correction. The selection analyses retained the same donor population under thresholds up to 30 cells, while changing the tissue mix. Thus they assess site sampling and precision rather than repair loss of entire patients. Pooling counts across matched sites also changes the weighting relative to equal-site program summaries and can reflect shifts in cellular composition. Batch-overlap limits, module curation, low-cell sensitivity estimates and untested selection mechanisms prevent a claim that the null is established for the full biological process.

Future studies would need a larger longitudinal cohort with balanced tissue sites and standardized processing, patient-level treatment and timing data, and direct functional measurements in sorted intestinal monocytes and macrophages, such as extracellular flux analysis, stable-isotope tracing or spatial metabolomics, to test whether metabolic reprogramming accompanies remission.

## Conclusion

In 15 patients with CD treated with adalimumab, the tested metabolic transcriptional programs, donor pseudobulk genes and curated metabolic transcript modules showed no remission contrast that survived their specified multiple-testing corrections. Batch-stratified and biopsy-selection analyses qualify the precision and generalizability of this result. Nonsignificance does not establish equivalence or absence of biological effects. Direct metabolic measurements, additional patients and fuller clinical metadata remain necessary to evaluate metabolic remodeling beyond these RNA-based observations.

## Supporting information

S1 Appendix Extended methods, statistical audit and supplementary results. Data integrity, reproduction, statistical-method audit, trajectory, scoring, positive-control, cohort-robustness and external-dataset analyses in detail.
(DOCX)

S1 Table Data-integrity checks. All 47 programmatic checks of the source files, matrix, identifiers, embeddings and metadata, with checksums.
(XLSX)

S2 Table Gene-set audit. Source, membership, mapping and scored genes for each program, and all pairwise overlaps.
(XLSX)

S3 Table Trajectory analyses. Official Slingshot lineages, cluster composition, patient coverage, branch-specific results, method comparisons, Palantir and metabolic-gene-free orderings.
(XLSX)

S4 Table Scoring analyses. UCell patient-level results for all families, rank-cutoff sensitivity, random gene-set reference, scoring-method comparison and gene-level panel.
(XLSX)

S5 Table Positive controls. Identity markers, trajectory control, published-benchmark tests, MASC reproduction, benchmark specification grid and literature marker programs.
(XLSX)

S6 Table Patient influence. Leave-one-patient-out estimates and exact P values for the 14 primary endpoints.
(XLSX)

S7 Table Detection limits and interval behavior. Empirical power, minimum detectable effects, family-level power, interval coverage, type I error and prospective sample sizes.
(XLSX)

S8 Table Alternative models. Patient-level alternative models, cell-level mixed models and dependence-aware multiplicity adjustment.
(XLSX)

S9 Table Site, support, shape and composition analyses. Terminal-ileum restriction, common pseudotime support, nonlinear summaries, within-state analyses and composition decomposition.
(XLSX)

S10 Table Myeloid recovery, selection and batch. Biopsy-level cell counts, recovery and composition analyses, eligible versus excluded pairs and batch structure.
(XLSX)

S11 Table Independent dataset screening and external check. Candidate datasets with reasons for acceptance or rejection, and GSE214695 program gradients.
(XLSX)

S12 Table Ulcerative colitis occupancy values. Individual patient values from the original exploratory ulcerative colitis occupancy analysis, shown descriptively without inference.
(XLSX)

S13 Table Batch diagnostics and conditional tests. Patient and pair counts, design identifiability, 40-allocation conditional tests, within-batch tests and patient/batch omission results.
(XLSX)

S14 Table Donor pseudobulk genes and metabolic transcript modules. Raw pseudobulk counts, library metadata, all gene tests, 17 curated module memberships, all 34 module tests per model and separate limma voom results.
(XLSX)

S15 Table Biopsy-selection sensitivity. All 26 site-pair changes, cell-threshold tests and explicitly hypothetical excluded-site sensitivity scenarios.
(XLSX)

S1 Fig Official Slingshot lineages. Lineages inferred with annotated, Louvain and k-means clusters on the released Harmony coordinates.
(TIF)

S2 Fig Comparison of pseudotime orderings. Distribution of Slingshot pseudotime by state and its relation to diffusion and geodesic pseudotime.
(TIF)

S3 Fig Marker expression along Slingshot pseudotime. Row-standardized expression of monocyte, macrophage and inflammatory markers by pseudotime decile.
(TIF)

S4 Fig Program profiles along Slingshot pseudotime. Descriptive mean program scores by outcome group and time point.
(TIF)

S5 Fig UCell sensitivity. Program-level contrasts by scoring method, agreement with original scores and comparison with size-matched random gene sets.
(TIF)

S6 Fig Composition and pseudotime support. Kitagawa decomposition of program-level change and per-biopsy pseudotime support.
(TIF)

S7 Fig Interval coverage and type I error. Simulated coverage of interval methods and type I error of unstudentized and studentized exact permutation tests.
(TIF)

S8 Fig Partial external check in GSE214695. Within-donor program gradients along diffusion pseudotime and the marker axis, compared with TAURUS.
(TIF)

S9 Fig Batch and donor influence. Change in adjusted program coefficients after omission of each patient or sequencing batch.
(TIF)

S10 Fig Complete metabolic module sensitivity. Directional and mixed-test adjusted P values for all 17 modules in all four model specifications.
(TIF)

## References

[1] Martin JC, Chang C, Boschetti G, Ungaro R, Giri M, Grout JA, et al. Single-cell analysis of crohn’s disease lesions identifies a pathogenic cellular module associated with resistance to anti-TNF Therapy. Cell. 2019;178(6):1493-1508.e20. doi: 10.1016/j.cell.2019.08.008 31474370 PMC7060942

[2] Thomas T, Friedrich M, Rich-Griffin C, Pohin M, Agarwal D, Pakpoor J, et al. A longitudinal single-cell atlas of anti-tumour necrosis factor treatment in inflammatory bowel disease. Nat Immunol. 2024;25(11):2152–65. doi: 10.1038/s41590-024-01994-8 39438660 PMC11519010

[3] Smillie CS, Biton M, Ordovas-Montanes J, Sullivan KM, Burgin G, Graham DB, et al. Intra- and inter-cellular rewiring of the human colon during ulcerative colitis. Cell. 2019;178(3):714-730.e22. doi: 10.1016/j.cell.2019.06.029 31348891 PMC6662628

[4] Friedrich M, Pohin M, Jackson MA, Korsunsky I, Bullers SJ, Rue-Albrecht K, et al. IL-1-driven stromal-neutrophil interactions define a subset of patients with inflammatory bowel disease that does not respond to therapies. Nat Med. 2021;27(11):1970–81. doi: 10.1038/s41591-021-01520-5 34675383 PMC8604730

[5] Bain CC, Bravo-Blas A, Scott CL, Perdiguero EG, Geissmann F, Henri S, et al. Constant replenishment from circulating monocytes maintains the macrophage pool in the intestine of adult mice. Nat Immunol. 2014;15(10):929–37. doi: 10.1038/ni.2967 25151491 PMC4169290

[6] Bain CC, Schridde A. Origin, differentiation, and function of intestinal macrophages. Front Immunol. 2018;9:2733. doi: 10.3389/fimmu.2018.02733 PMC6277706 30538701

[7] Bujko A, Atlasy N, Landsverk OJB, Richter L, Yaqub S, Horneland R, et al. Transcriptional and functional profiling defines human small intestinal macrophage subsets. J Exp Med. 2018;215(2):441–58. doi: 10.1084/jem.20170057 29273642 PMC5789404

[8] Kong L, Pokatayev V, Lefkovith A, Carter GT, Creasey EA, Krishna C, et al. The landscape of immune dysregulation in Crohn’s disease revealed through single-cell transcriptomic profiling in the ileum and colon. Immunity. 2023;56(2):444-458.e5. doi: 10.1016/j.immuni.2023.01.002 36720220 PMC9957882

[9] Garrido-Trigo A, Corraliza AM, Veny M, Dotti I, Melón-Ardanaz E, Rill A, et al. Macrophage and neutrophil heterogeneity at single-cell spatial resolution in human inflammatory bowel disease. Nat Commun. 2023;14(1):4506. doi: 10.1038/s41467-023-40156-6 37495570 PMC10372067

[10] O’Neill LAJ, Kishton RJ, Rathmell J. A guide to immunometabolism for immunologists. Nat Rev Immunol. 2016;16(9):553–65. doi: 10.1038/nri.2016.70 27396447 PMC5001910

[11] Van den Bossche J, O’Neill LA, Menon D. Macrophage immunometabolism: where are we (going)? Trends Immunol. 2017;38(6):395–406. doi: 10.1016/j.it.2017.03.001 28396078

[12] Zeng Z, Cheng S, Li X, Liu H, Lin J, Liang Z, et al. Glycolytic Activation of CD14+ intestinal macrophages contributes to the inflammatory responses via exosomal membrane tumor necrosis factor in crohn’s disease. Inflamm Bowel Dis. 2024;30(1):90–102. doi: 10.1093/ibd/izad117 37406645

[13] Koelink PJ, Bloemendaal FM, Li B, Westera L, Vogels EWM, van Roest M, et al. Anti-TNF therapy in IBD exerts its therapeutic effect through macrophage IL-10 signalling. Gut. 2020;69(6):1053–63. doi: 10.1136/gutjnl-2019-318264 31506328 PMC7282553

[14] Zhang Y-F, Shi TT, Lin Y-L, Zhu Y-T, Lin S, Fang T-Y. Macrophage metabolic reprogramming in inflammatory bowel diseases: from pathogenesis to therapy. J Inflamm Res. 2025;18:11821–39. doi: 10.2147/JIR.S534447 40901027 PMC12399898

[15] Yang Y, Zhu H. Macrophage dysregulation in inflammatory bowel disease: cellular heterogeneity, pathogenic mechanism, and treatment. Front Immunol. 2026;17:1837255. doi: 10.3389/fimmu.2026.1837255 42253951 PMC13233708

[16] Zheng HB, Doran BA, Kimler K, Yu A, Tkachev V, Niederlova V, et al. Concerted changes in the pediatric single-cell intestinal ecosystem before and after anti-TNF blockade. Elife. 2026;12:RP91792. doi: 10.7554/eLife.91792 42678060 PMC13533482

[17] Korsunsky I, Millard N, Fan J, Slowikowski K, Zhang F, Wei K, et al. Fast, sensitive and accurate integration of single-cell data with Harmony. Nat Methods. 2019;16(12):1289–96. doi: 10.1038/s41592-019-0619-0 31740819 PMC6884693

[18] Wolf FA, Angerer P, Theis FJ. SCANPY: large-scale single-cell gene expression data analysis. Genome Biol. 2018;19(1):15. doi: 10.1186/s13059-017-1382-0 29409532 PMC5802054

[19] Haghverdi L, Büttner M, Wolf FA, Buettner F, Theis FJ. Diffusion pseudotime robustly reconstructs lineage branching. Nat Methods. 2016;13(10):845–8. doi: 10.1038/nmeth.3971 27571553

[20] Saelens W, Cannoodt R, Todorov H, Saeys Y. A comparison of single-cell trajectory inference methods. Nat Biotechnol. 2019;37(5):547–54. doi: 10.1038/s41587-019-0071-9 30936559

[21] Street K, Risso D, Fletcher RB, Das D, Ngai J, Yosef N, et al. Slingshot: cell lineage and pseudotime inference for single-cell transcriptomics. BMC Genomics. 2018;19(1):477. doi: 10.1186/s12864-018-4772-0 29914354 PMC6007078

[22] Setty M, Kiseliovas V, Levine J, Gayoso A, Mazutis L, Pe’er D. Characterization of cell fate probabilities in single-cell data with Palantir. Nat Biotechnol. 2019;37(4):451–60. doi: 10.1038/s41587-019-0068-4 30899105 PMC7549125

[23] Liberzon A, Birger C, Thorvaldsdóttir H, Ghandi M, Mesirov JP, Tamayo P. The Molecular Signatures Database (MSigDB) hallmark gene set collection. Cell Syst. 2015;1(6):417–25. doi: 10.1016/j.cels.2015.12.004 26771021 PMC4707969

[24] Gillespie M, Jassal B, Stephan R, Milacic M, Rothfels K, Senff-Ribeiro A. The reactome pathway knowledgebase 2022. Nucleic Acids Res. 2022;50(D1):D687–92. doi: 10.1093/nar/gkab1028 PMC8689983 34788843

[25] Kanehisa M, Furumichi M, Sato Y, Kawashima M, Ishiguro-Watanabe M. KEGG for taxonomy-based analysis of pathways and genomes. Nucleic Acids Res. 2023;51(D1):D587–92. doi: 10.1093/nar/gkac963 36300620 PMC9825424

[26] Andreatta M, Carmona SJ. UCell: Robust and scalable single-cell gene signature scoring. Comput Struct Biotechnol J. 2021;19:3796–8. doi: 10.1016/j.csbj.2021.06.043 34285779 PMC8271111

[27] Goeman JJ, Bühlmann P. Analyzing gene expression data in terms of gene sets: methodological issues. Bioinformatics. 2007;23(8):980–7. doi: 10.1093/bioinformatics/btm051 17303618

[28] Benjamini Y, Hochberg Y. Controlling the false discovery rate: a practical and powerful approach to multiple testing. J R Stat Soc Series B Stat Methodol. 1995;57(1):289–300. doi: 10.1111/j.2517-6161.1995.tb02031.x

[29] Westfall PH, Young SS. Resampling-based multiple testing: examples and methods for p-value adjustment. New York: Wiley. 1993.

[30] Li J, Ji L. Adjusting multiple testing in multilocus analyses using the eigenvalues of a correlation matrix. Heredity (Edinb). 2005;95(3):221–7. doi: 10.1038/sj.hdy.6800717 16077740

[31] Janssen A. Studentized permutation tests for non-i.i.d. hypotheses and the generalized Behrens-Fisher problem. Stat Probab Lett. 1997;36(1):9–21. doi: 10.1016/S0167-7152(97)00043-6

[32] Chung E, Romano JP. Exact and asymptotically robust permutation tests. Ann Stat. 2013;41(2):484–507. doi: 10.1214/13-AOS1090

[33] Lakens D. Equivalence tests: a practical primer for t tests, correlations, and meta-analyses. Soc Psychol Personal Sci. 2017;8(4):355–62. doi: 10.1177/1948550617697177 28736600 PMC5502906

[34] Kitagawa EM. Components of a difference between two rates. J Am Stat Assoc. 1955;50(272):1168–94. doi: 10.1080/01621459.1955.10501299

[35] Bates D, Machler M, Bolker B, Walker S. Fitting linear mixed-effects models using lme4. J Stat Softw. 2015;67(1):1–48. doi: 10.18637/jss.v067.i01

[36] Kuznetsova A, Brockhoff PB, Christensen RHB. lmerTest package: tests in linear mixed effects models. J Stat Softw. 2017;82(13):1–26. doi: 10.18637/jss.v082.i13

[37] Chen Y, Chen L, Lun ATL, Baldoni PL, Smyth GK. edgeR v4: powerful differential analysis of sequencing data with expanded functionality and improved support for small counts and larger datasets. Nucleic Acids Res. 2025;53(2):gkaf018. doi: 10.1093/nar/gkaf018 39844453 PMC11754124

[38] Ritchie ME, Phipson B, Wu D, Hu Y, Law CW, Shi W, et al. limma powers differential expression analyses for RNA-sequencing and microarray studies. Nucleic Acids Res. 2015;43(7):e47. doi: 10.1093/nar/gkv007 25605792 PMC4402510

[39] Fonseka CY, Rao DA, Teslovich NC, Korsunsky I, Hannes SK, Slowikowski K, et al. Mixed-effects association of single cells identifies an expanded effector CD4+ T cell subset in rheumatoid arthritis. Sci Transl Med. 2018;10(463):eaaq0305. doi: 10.1126/scitranslmed.aaq0305 30333237 PMC6448773

