# Supplementary material for: No detectable remission-specific remodeling of intestinal monocyte and macrophage metabolic transcriptional programs after adalimumab in Crohn’s disease: A patient-level reanalysis of a longitudinal single-cell atlas
Source: PLoS One. 2026 Nov 11;21(11):e0356953. doi: 10.1371/journal.pone.0356953 (PMC13754127; doi:10.1371/journal.pone.0356953)
Supplement: Supplementary file 1 [file pone.0356953.s001.docx]

# S1 Appendix. Extended methods, statistical audit and supplementary results

This appendix accompanies the revised manuscript PONE-D-26-39679. All numbers are generated from executed scripts; file names refer to the reproducibility archive and to S1–S15 Tables.

## 1. Data integrity and exact reproduction

47 of 47 programmatic checks passed (S1 Table). The archived release 0.1.0 scripts were rerun unmodified; all 27 archived tables were regenerated with a maximum absolute difference of 0. The count matrix stores column indices unsorted within rows; the R import uses sparseMatrix(), and alignment of cells, genes, library sizes and Harmony coordinates between Python and R was asserted before analysis. Healthy-control ages differ between the h5ad and source Supplementary Table 1B (not used).

## 2. Statistical-method audit

Percentile bootstrap intervals undercovered (mean 0.893, minimum 0.828); Welch intervals achieved 0.940 and are reported as primary. With unequal variances, the unstudentized exact test had type I error 0.019 to 0.140; the studentized exact test reduced the maximum to 0.095. The effective number of independent endpoints was 6; the exact single-step maximum-statistic adjusted P was at least 0.980. Simulations resampled observed residuals within outcome groups; 400 (bootstrap, Welch) or 150 (permutation inversion) datasets per endpoint for coverage, 4,000 for type I error, and 2,000 per effect size for power (S7 Table).

## 3. Gene sets

795 memberships; 710 distinct source genes; 707 mapped; 694 with nonzero variance among monocytes/macrophages; 67 genes in two or more programs (S2 Table).

| **Program** | **Library** | **Source** | **Mapped** | **Scored (original)** | **Scored (UCell)** | **Median detection** |
| --- | --- | --- | --- | --- | --- | --- |
| Glycolysis | MSigDB_Hallmark_2020 | 200 | 199 | 196 | 199 | 0.042 |
| Oxidative phosphorylation | MSigDB_Hallmark_2020 | 200 | 200 | 200 | 200 | 0.211 |
| Fatty acid metabolism | MSigDB_Hallmark_2020 | 158 | 157 | 151 | 157 | 0.075 |
| Cholesterol homeostasis | MSigDB_Hallmark_2020 | 74 | 73 | 73 | 73 | 0.059 |
| TCA cycle | Reactome_2022 | 21 | 21 | 21 | 21 | 0.107 |
| Pentose phosphate pathway | Reactome_2022 | 14 | 14 | 13 | 14 | 0.077 |
| Lysosome | KEGG_2021_Human | 128 | 128 | 125 | 128 | 0.120 |

## 4. Trajectory analyses

| **Run** | **Lineage** | **Path (majority state of each cluster)** | **Cells (weight >= 0.5)** | **Spearman with DPT** |
| --- | --- | --- | --- | --- |
| primary_annotated_HARMONY50 | Lineage1 | S100A8/A9hi mono -> TNFhi IL6+ mono -> C1Qhi IL1Blo macro -> C1Qhi IL1Bhi macro | 15707 | 0.35 |
| louvain_HARMONY20 | Lineage1 | S100A8/A9hi mono -> S100A8/A9hi mono -> TNFhi IL6+ mono -> C1Qhi IL1Blo macro -> C1Qhi IL1Blo macro -> C1Qhi IL1Blo macro | 11535 | 0.96 |
| louvain_HARMONY20 | Lineage2 | S100A8/A9hi mono -> S100A8/A9hi mono -> C1Qhi IL1Bhi macro -> C1Qhi IL1Bhi macro | 9497 | 0.97 |
| kmeans10_HARMONY20 | Lineage1 | S100A8/A9hi mono -> S100A8/A9hi mono -> TNFhi IL6+ mono -> C1Qhi IL1Blo macro -> C1Qhi IL1Blo macro -> C1Qhi IL1Bhi macro -> C1Qhi IL1Bhi macro | 12825 | 0.47 |
| kmeans10_HARMONY20 | Lineage2 | S100A8/A9hi mono -> S100A8/A9hi mono -> TNFhi IL6+ mono -> C1Qhi IL1Blo macro -> C1Qhi IL1Blo macro -> C1Qhi IL1Blo macro | 9016 | 0.97 |
| kmeans10_HARMONY20 | Lineage3 | S100A8/A9hi mono -> S100A8/A9hi mono -> S100A8/A9hi mono | 6187 | 0.44 |

Slingshot was run on a SingleCellExperiment constructed from the h5ad counts with the released 50-dimensional Harmony coordinates as the reduced dimension (approx_points = 150). Annotation-free clusters were computed with bluster (Louvain on a 30-nearest-neighbour graph; k-means with k = 10 and 10 starts) on the first 20 Harmony dimensions, with the start cluster defined as the cluster containing the outcome-blind DPT root cell. Palantir used 10 diffusion components, 30 neighbours and 1,200 waypoints from the same root cell.

## 5. Positive controls

MASC was refitted as glmer(state ~ contrast + age + sex + site + disease duration + percent mitochondrial reads + (1|donor) + (1|donor:biopsy)), family binomial, with likelihood-ratio tests against the model without the contrast. The published populations were identified by matching published cell counts exactly (for example 1,164 C1Q-high IL1B-low cells in 26 paired pretreatment biopsies). Patient-level benchmarks used pseudobulk means and exact tests (S5 Table).

## 6. Additional robustness checks from the adversarial review

Batch-matched subset (batches 1 and 3; 4 versus 5 patients): minimum adjusted P 0.22. Ambient epithelial transcripts in myeloid droplets increased in all patients (P = 0.0005) without an outcome difference. Mitochondrial-adjusted pentose phosphate decrease q = 0.048. Across 481 exploratory remission-contrast tests, 10 had nominal P <0.05 (S8 Table).

## 7. Software

Python 3.12.9 with AnnData 0.13.2, Scanpy 1.12.3, NumPy 2.3.5, SciPy 1.18.0, pandas 3.0.5, statsmodels 0.14.6, scikit-learn 1.7.2 and GSEApy 1.3.1; Palantir 1.4.1 with harmonypy 2.1.0 and pandas 2.3.3 in a separate environment; R 4.6.1 (aarch64-apple-darwin) with Bioconductor 3.23: slingshot 2.20.0, UCell 2.16.0, SingleCellExperiment 1.34.0, SummarizedExperiment 1.42.0, TrajectoryUtils 1.20.0, princurve 2.1.6, bluster 1.22.0, BiocParallel 1.46.0, rhdf5 2.56.1, Matrix 1.7.6, DelayedMatrixStats 1.34.0, lme4 2.0.6 and lmerTest 3.2.1. Full session information is in the reproducibility archive.

# Final targeted analyses

The following section records the final targeted computational round. It supersedes any earlier interpretation equating power-based detection thresholds with evidence that an effect is absent. Earlier analyses retain their original provenance and were not all rerun in this round.

## Exploratory batch diagnostics and donor pseudobulk analyses

We added the following analyses after peer review, before inspecting their new results. Batch diagnostics counted independent patients by outcome and sequencing batch, checked design rank and the residual variation in outcome after batch adjustment, and enumerated outcome-label permutations within each batch while preserving its remission count. The statistic was the remission coefficient from regression of patient-level post-minus-pre change on outcome and batch indicators. This conditional test requires exchangeability of patients within batch under the null; outcome was observational and was not randomized. The 14 program endpoints formed one Benjamini–Hochberg family for each specification. We examined the shared batches, each informative batch separately, and omission of individual patients or batches (S13 Table).

For transcriptome-wide analysis, we summed raw integer monocyte/macrophage counts within each patient and time point across the same eligible tissue sites, producing 30 pseudobulk libraries from 15 patients. Duplicate gene symbols, if any, were summed, with Ensembl-to-symbol mappings retained. We applied edgeR filterByExpr using the four outcome-by-time groups, TMM library normalization, robust dispersion estimation and quasi-likelihood generalized linear models. Patient fixed effects accounted for paired observations; post-treatment and post-treatment-by-remission terms estimated the difference in change. Batch-adjusted models replaced the common post-treatment term with batch-specific post-treatment terms. Batch main effects were absorbed by patient effects because every patient belonged to one batch. We checked full design rank and retained residual degrees of freedom. Sensitivity models used the nine patients in batches containing both outcomes and all 26 available CD site pairs. Sites and cells were never counted as independent donors (Table 5; S14 Table).

Seventeen explicitly curated enzyme or complex transcript modules resolved glycolysis, pyruvate entry, the TCA cycle, oxidative and nonoxidative pentose phosphate reactions, respiratory complexes I–IV, ATP synthase, fatty-acid synthesis and oxidation, cholesterol synthesis and transport, and lysosomal acidification and hydrolases. Membership was fixed in the analysis script before model fitting and is fully listed in S14 Table. These targeted modules do not constitute an exhaustive database enrichment screen. Using the same paired design, limma voom and fry tested directional and mixed-direction module responses; all 34 tests were adjusted together within each model. Genes were adjusted across all expressed genes tested in that model. A separate limma voom gene analysis assessed agreement with edgeR. Sensitivity specifications were reported completely and were not selected by significance. Neither transcript modules nor model coefficients measure enzyme activity or metabolic flux.

## Cohort-selection sensitivity analyses

The selection rule excluded site pairs rather than whole patients: all 15 paired CD patients retained at least one eligible site. We re-estimated equal-site, within-patient program changes using thresholds of 1, 5, 10, 20, 30 and 50 cells at each time point, including descriptive low-cell estimates for all 26 pairs. The original global outcome-blind continuum and scoring definitions were held fixed, isolating the effect of site inclusion. Slopes required at least three cells and three distinct pseudotimes per biopsy; all actual tested-pair counts were retained. Each threshold was tested with and without batch adjustment and corrected across 14 endpoints. Low-cell estimates have greater sampling uncertainty and are sensitivity analyses.

We additionally varied unobserved reliable changes in excluded sites under a stated selection assumption: each excluded site was assigned its donor’s eligible-site mean change plus a common shift of −2, −1, 0, 1 or 2 pooled donor standard deviations. Each scenario was re-averaged within patient and batch-stratified. These values are hypothetical sensitivity parameters rather than measured effects or clinical imputations. Finite sensitivity ranges are conditional on their assumptions; the data do not provide assumption-free bounds. Inclusion weighting was not used because the cell-threshold rule is deterministic and cannot restore missing reliable site measurements (S15 Table).

## Batch confounding and transcriptome-wide donor analyses

Batch 1 contained 1 remission and 3 nonremission patients, batch 2 contained 4 remission patients, batch 3 contained 3 remission and 2 nonremission patients, and batch 4 contained 2 remission patients. Batch explained 41.5% of the patient-level variation in outcome. The patient-change regression had full rank (5 columns), but within-batch comparisons were informed by only nine patients in batches 1 and 3. There were 40 conditional label allocations. The smallest batch-stratified P value was 0.025 and the smallest adjusted P across 14 endpoints was 0.350; the shared-batch analysis gave the same coefficient and conditional P values. No within-batch result survived correction. Patient and batch omission analyses are reported in full in S13 Table. Time-invariant additive batch effects cancel in paired changes, whereas batch-dependent changes remain a possible confounder.

The primary pseudobulk analysis tested 7092 expressed genes. No gene survived Benjamini–Hochberg correction in the unadjusted model (minimum adjusted P 0.298) or in the model allowing batch-specific post-treatment changes (minimum adjusted P 0.894). The adjusted model had rank 20 of 20 and 10 residual degrees of freedom. Models restricted to shared batches (7739 genes; 6 residual degrees of freedom) and including all 26 site pairs (7021 genes; 10 residual degrees of freedom) also yielded no corrected gene findings. The independent limma voom model detected no gene at adjusted P <0.05 (minimum adjusted P 0.421); log-fold-change estimates correlated with edgeR estimates (Spearman ρ = 0.965). These analyses extend resolution beyond the previous 31-gene panel but remain limited by the small number of independent patients (Fig 6; Table 5; S14 Table).

None of the 17 metabolic transcript modules was significant after joint correction of directional and mixed tests in any model. In the primary batch-adjusted model, upper glycolysis had the smallest directional P value (0.00393), but its adjusted P across all 34 module tests was 0.134. This nominal observation is exploratory and does not establish a subpathway effect. All module memberships, expressed-gene counts and both test types are provided in S14 Table and S10 Fig.

## Sensitivity to biopsy-pair selection

Seven of 26 CD site pairs failed the primary 30-cell rule. All seven were colonic and six belonged to remission patients; no paired CD patient was excluded completely. Thresholds of 1, 5, 10, 20, 30 and 50 retained 26, 25, 25, 20, 19 and 14 pairs, respectively, from 15, 15, 15, 15, 15 and 12 patients. None of the 14 contrasts survived correction at any threshold, with or without batch adjustment. The all-pair batch-adjusted minimum adjusted P was 0.850 compared with 0.350 at the primary threshold. The hypothetical excluded-site scenarios from −2 to +2 SD yielded no corrected contrast (smallest adjusted P 0.117). These checks show sensitivity over the evaluated rules and assumptions; they do not remove site-selection bias or recover reliable measurements from sparsely sampled sites (Fig 6; S15 Table).

## Functional measurements and clinical information

No functional metabolic experiment, new patient recruitment, measured flux or additional clinical metadata was obtained. All supplementary gene and module results are transcriptomic. Individual dose histories, treatment intervals and concomitant medications remain unavailable for adjustment. No investigator outreach or external submission was performed.
